# ARID3B Induces Tumor Necrosis Factor Alpha Mediated Apoptosis While a Novel ARID3B Splice Form Does Not Induce Cell Death

**DOI:** 10.1371/journal.pone.0042159

**Published:** 2012-07-31

**Authors:** Stancy Joseph, Victoria E. Deneke, Karen D. Cowden Dahl

**Affiliations:** 1 Department of Biochemistry and Molecular Biology, Indiana University School of Medicine, South Bend, Indiana, United States of America; 2 Department of Chemistry and Biochemistry and Eck Institute for Global Health, Notre Dame University, Notre Dame, Indiana, United States of America; 3 Indiana University Melvin and Bren Simon Cancer Center, Indianapolis, Indiana, United States of America; 4 Department of Engineering, University of Notre Dame, Notre Dame, Indiana, United States of America; University of Nebraska Medical Center, United States of America

## Abstract

Alternative splicing is a common occurrence in many cancers. Alternative splicing is linked with decreased apoptosis and chemoresistance in cancer cells. We previously demonstrated that ARID3B, a member of the AT-rich interactive domain (ARID) family of DNA binding proteins, is overexpressed in ovarian cancer. Therefore we wanted to assess the effect of ARID3B splice forms on cell viability. We identified a novel splice form of the ARID3B gene (designated as ARID3B Sh), which lacks the C-terminal exons 5–9 present in the full-length isoform (ARID3B Fl). ARID3B Fl is expressed in a variety of cancer cell lines. Expression of ARID3B Sh varied by cell type, but was highly expressed in most ovarian cancer lines. ARID3B is modestly transcriptionally activated by epidermal growth factor receptor (EGFR) signaling through the PEA3 transcription factor. We further found that ARID3B Fl is predominantly nuclear but is also present at the plasma membrane and in the cytosol. Endogenous ARID3B Sh is present in nuclear fractions, yet, when overexpressed ARID3B Sh accumulates in the cytosol and membrane fractions. The differential localization of these isoforms suggests they have different functions. Importantly, ARID3B Fl overexpression results in upregulation of pro-apoptotic BIM and induces Tumor Necrosis Factor alpha (TNFα) and TNF-related apoptosis inducing ligand (TRAIL) induced cell death. The ARID3B Fl-induced genes include TNFα, TRAIL, TRADD, TNF-R2, Caspase 10 and Caspase 7. Interestingly, ARID3B Sh does not induce apoptosis or expression of these genes. ARID3B Fl induces death receptor mediated apoptosis while the novel splice form ARID3B Sh does not induce cell death. Therefore alternative splice forms of ARID3B may play different roles in ovarian cancer progression.

## Introduction

Ovarian cancer represents the most lethal gynecological malignant disease in the United States. According to the American Cancer Society [1], if diagnosed at the localized stage, the 5-year survival rate is 94%; however, only 15% of all cases are detected at this stage. The majority of cases of ovarian cancer (62%) are diagnosed with distant metastases [1]. For these women the 5-year survival rate is 28% [1], therefore determining the unique genetic programming that drives ovarian cancer progression is key in diagnosing and treating this disease. We previously identified ARID3B as a target of miR-125a, a microRNA that is under expressed in ovarian cancer [2], [3], [4]. However, the function of ARID3B is relatively unknown. ARID3B belongs to the ARID family of proteins. The ARID family of transcriptional regulators is a conserved group of DNA binding proteins that regulates gene expression [5], [6]. ARID proteins harbor a distinctive DNA-binding domain, the AT-rich interactive domain (ARID). Proteins of this family have been implicated in regulation of cell cycle, gene expression, differentiation, embryonic development, chromatin-remodeling and transcriptional regulation [6], [7], [8], [9]. *ARID3B/BDP/Dril2* is the third factor of the ARID3 subfamily. ARID3B is most similar to its paralogue ARID3A, however ARID3B and ARID3A are not closely related outside of the ARID, extended ARID, and REKLES domains [5], [7], [8], [10]. *In vitro* translated ARID3B binds to the same region of DNA as ARID3A near the immunoglobulin heavy chain enhancer called a matrix attachment region. ARID3A is primarily expressed in B-lymphocytes in adults [8], [10], [11]. In contrast ARID3B is much more widely expressed in the adult and is particularly highly expressed in stratified epithelium and secretory cells (data not shown). Unlike ARID3A, which actively shuttles between the nucleus and cytoplasm, ARID3B was shown to localize exclusively to the nucleus when overexpressed in B-cell lines and in Cos7 cells [10], [12]. ARID3B enhances the nuclear localization of ARID3A by interacting with its REKLES β domain [10]. REKLES β mediated ARID3A-ARID3B interaction blocks nucleocytoplasmic shuttling of ARID3A by interfering with the nuclear export activity [10]. However, ARID3A is incapable of re-localizing ARID3B, suggesting a dominant effect for ARID3B on the cellular localization of both proteins [10]. Thus, ARID3B could be key regulator in ARID3A function by regulating cellular localization in B cells. Since ARID3B is expressed more broadly than ARID3A it likely has other functions than its regulation of ARID3A.

*ARID3B* is essential during embryonic development [13], [14], [15]. Deletion of *Arid3b* in mice leads to embryonic lethality, poor angiogenesis, limb bud defects, and craniofacial and cardiovascular malformations [13], [14], [15]. Aberrant expression of ARID3B is found in malignant tumors. ARID3B is expressed in human neuroblastoma cell lines and in stage IV neuroblastoma, but not in stage I-III, indicating a possible role in the progression of malignant neuroblastoma [14]. Importantly, ARID3B is overexpressed in serous ovarian cancer [2]. However the function and regulation of ARID3B in cancer has not been fully evaluated.

Although little is known about the mechanisms that contribute to ovarian cancer progression, epidermal growth factor receptor (EGFR or ErbB1) is overexpressed in up to 70% of ovarian cancer [16], [17], [18]. The overexpression of EGFR in ovarian cancer correlates with poor prognosis [18], [19]. EGFR promotes tumor progression in a number of ways. EGFR not only regulates invasion through inducing genes such the ETS factor PEA3 [2], [20]; it also regulates alternative splicing [21]. Since both EGFR and ARID3B are overexpressed in ovarian cancer we wanted to assess if there was a causal relationship between these molecules and ascertain if ARID3B is EGFR-regulated in ovarian cancer cells.

Due to overexpression of ARID3B in ovarian cancer, we wanted to define the function and regulation of ARID3B in ovarian cancer cells. In this study we identified and validated an alternate splice form of ARID3B. We found that ARID3B isoforms are not significantly regulated by EGFR signaling. We have determined that the longer splice form, ARID3B Fl, induces expression of genes involved in TNFα/TRAIL signaling and BIM. Importantly, the novel splice form, ARID3B Sh (which is a shorter splice form), does not induce cell death. Our results suggest that regulating the splicing of ARID3B may be important in ovarian cancer progression.

## Results

### Identification of an Alternate Splice Form of ARID3B **(**ARID3B Sh)

**Figure 1:**
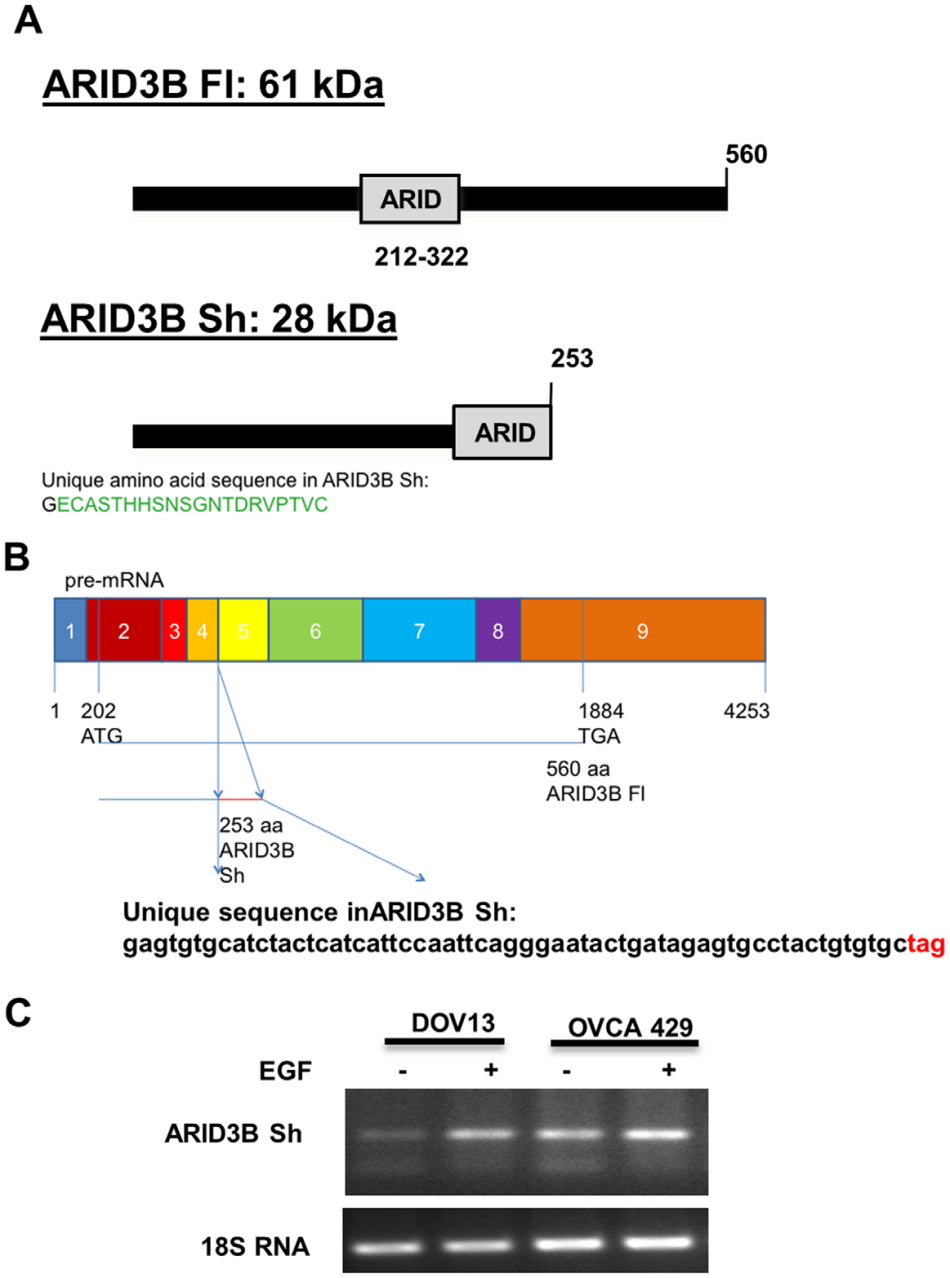
Identification of a novel alternative splice form of ARID3B (ARID3B Sh). **A**. Schematic representations of the proteins generated from the human ARID3B splice forms. ARID3B Sh contains a unique epitope from intron 4. **B**. Schematic representation of the human ARID3B splice forms pre-mRNA sequence. The unique mRNA sequence found in ARID3B Sh is depicted. **C**. The expression of ARID3B Sh was confirmed in ovarian cancer cell lines. The human ovarian cancer cell lines, OVCA 429 and DOV13, were serum starved (SS) for 24 h, treated with or without 20 nM EGF for an additional 24 h. RT-PCR analyses for ARID3B Sh and 18 s rRNA were performed.

In examining the ARID3B locus via the University of California Santa Cruz Genome Browser, multiple expressed sequence tags (ESTs) corresponding to an alternate splice form of ARID3B were reported. We refer to this alternate isoform as ARID3B Sh since this isoform is substantially smaller than the full-length transcript of ARID3B (ARID3B Fl) (Fig. 1A). ARID3B Sh is 253 amino acids containing exons 1–4 and a small unique sequence from intron 4 (Fig. 1B). This results in loss of 81% of the ARID DNA binding domain. To determine if ARID3B Sh is expressed in ovarian carcinoma cells we performed reverse transcribed polymerase chain reaction (RT-PCR). The ARID3B Sh splice form was verified using DOV13 and OVCA 429 ovarian cancer cells treated with or without EGF for 24 h. cDNA was generated and ARID3B Sh was amplified using primers specific to the 3′ end of ARID3B Sh. The RT-PCR product was observed at 158 bp (Fig. 1C). This data confirms the identification and expression of the novel alternative splice form of ARID3B, ARID3B Sh in ovarian cancer cells.

### ARID3B Splice Forms are Expressed in Cancer Cell Lines

**Figure 2:**
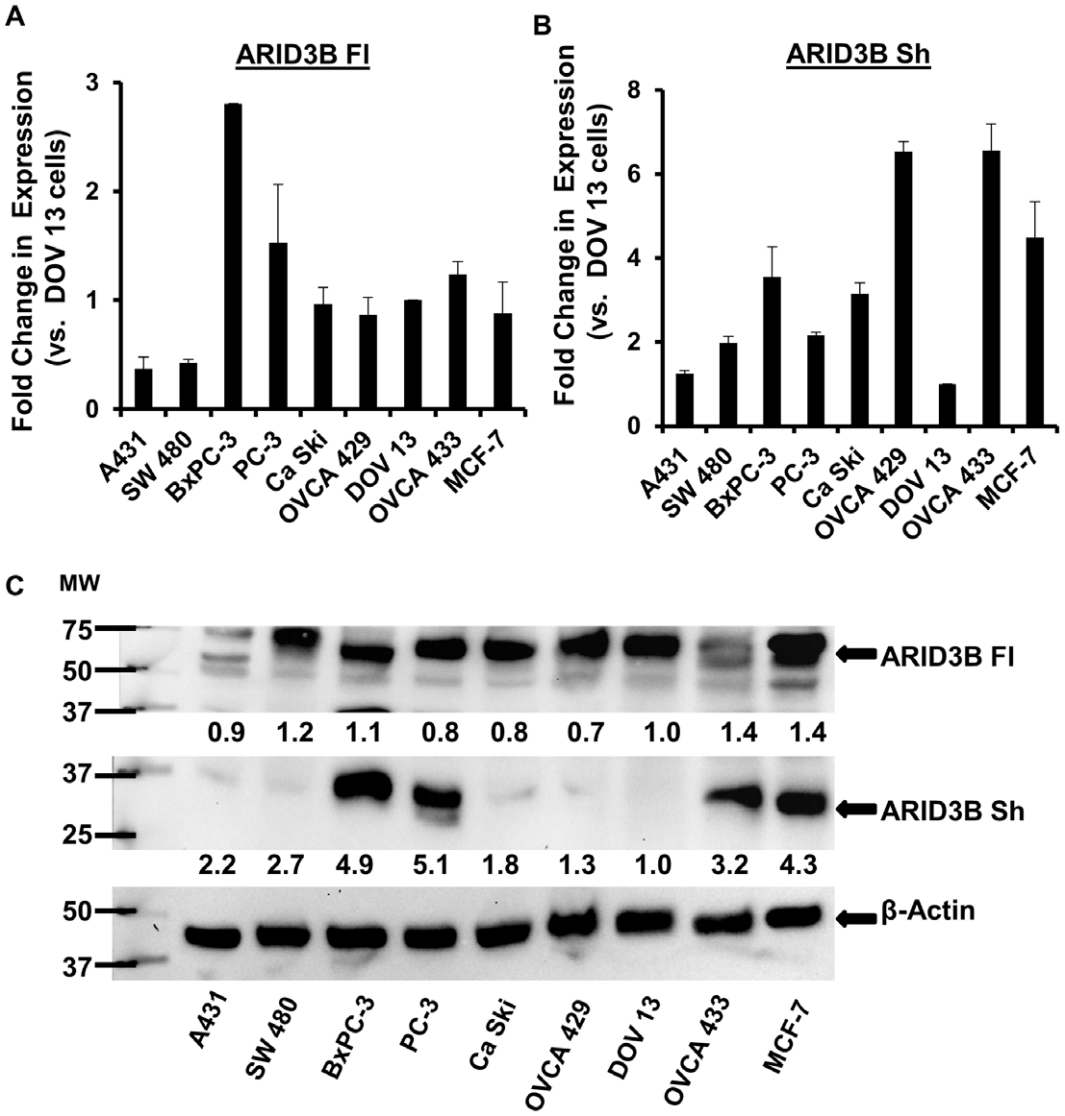
Expression of ARID3B splice forms in cancer cell lines. QRT-PCR for ARID3B Fl (**A**) and ARID3B Sh (**B**) was performed on total RNA isolated from the following cancer cell lines; A431 (skin), SW 480 (colon), BxPC-3 (pancreatic), PC-3 (prostate), Ca Ski (cervical), MCF-7 (breast), and three ovarian cancer cell lines, DOV13, OVCA 433 and OVCA 429. Results were normalized to 18 S rRNA expression and compared to the expression of ARID3B Fl or Sh in DOV13 ovarian cancer cells. Fold expression relative to DOV13 cells was expressed as the mean ± SEM of triplicate measurements. **C**. Western blot was performed for ARID3B and β-actin on cancer cell line lysates. Results were normalized to β-actin expression and compared to the expression of ARID3B Fl/Sh in DOV13 ovarian cancer cells. The densitometry evaluation of the western blot analyses for ARID3B Fl and ARID3B Sh were analyzed (value under blot). Fold expression relative to DOV13 cells was expressed as the mean of triplicate measurements.

ARID3B is overexpressed in ovarian cancer and neuroblastoma [2], [14], but these studies did not distinguish between ARID3B Fl and ARID3B Sh. To determine if ARID3B splice forms are expressed in cancer cell lines, we obtained nine cancer cell lines; A431 (skin), SW 480 (colon), BxPC-3 (pancreatic), PC-3 (prostate), Ca Ski (cervical), MCF-7 (breast), and three ovarian cancer cell lines, DOV13, OVCA 433 and OVCA 429. We examined the mRNA and protein expression of ARID3B Fl and ARID3B Sh. RNA was collected from each cell line, cDNA was generated and quantitative RT-PCR (QRT-PCR) was performed for ARID3B Fl, ARID3B Sh and 18 S rRNA (control) (data was normalized to DOV13 ovarian cancer cells) (Fig. 2A and 2B). The expression of both splice forms, ARID3B Fl and ARID3B Sh, was observed in all nine cancer cell lines. The mRNA expression of ARID3B Fl and ARID3B Sh (relative to DOV13 cell line) varied widely in the different cell types. The highest level of ARID3B Fl was observed in the BxPC-3 (pancreatic) cell line (2.8-fold increase over DOV13 cell line) (Fig. 2A) and the lowest level in A431 (skin) cell line (2.7-fold decrease compared to DOV13 cell line). The highest level of ARID3B Sh (Fig. 2B) was observed in the OVCA 429 and OVCA 433 (ovarian) cell lines (6.5- and 6.6-fold increase over DOV13 cell line, respectively) and the lowest level in the DOV13 (ovarian) cell line. Western blot analysis from whole cell lysates from the same cancer cell lines was performed for both splice forms. Validation of the antibody used for blotting both ARID3B isoforms is described in materials and methods. All of the cell lines expressed comparable levels of ARID3B Fl protein (Fig. 2C). Expression of ARID3B Sh was more variable. Although ARID3B Sh was expressed in all the cell lines, highest expression was observed in the BxPC-3, PC-3, MCF-7 and OVCA 433 cells compared to the other cell lines in the study (Fig. 2C). This finding indicates that both ARID3B splice forms are present in a variety of cancer cells lines and their expression varies by cell type. We also examined expression of ARID3B isoforms in a tissue blot from Imgenex that contained lysates from seven different serous papillary adenocarcinomas and their matching normal adjacent tissue. We found that ARID3B Fl was expressed in tumor tissue but not normal adjacent tissue (Figure S1). ARID3B Sh was also expressed in tumor tissue and in some normal adjacent lysates (Figure S1). Therefore both forms of ARID3B can be found in ovarian cancer cell lines and ovarian tumors.

### EGF Modestly Regulates Expression of ARID3B Isoforms

**Figure 3:**
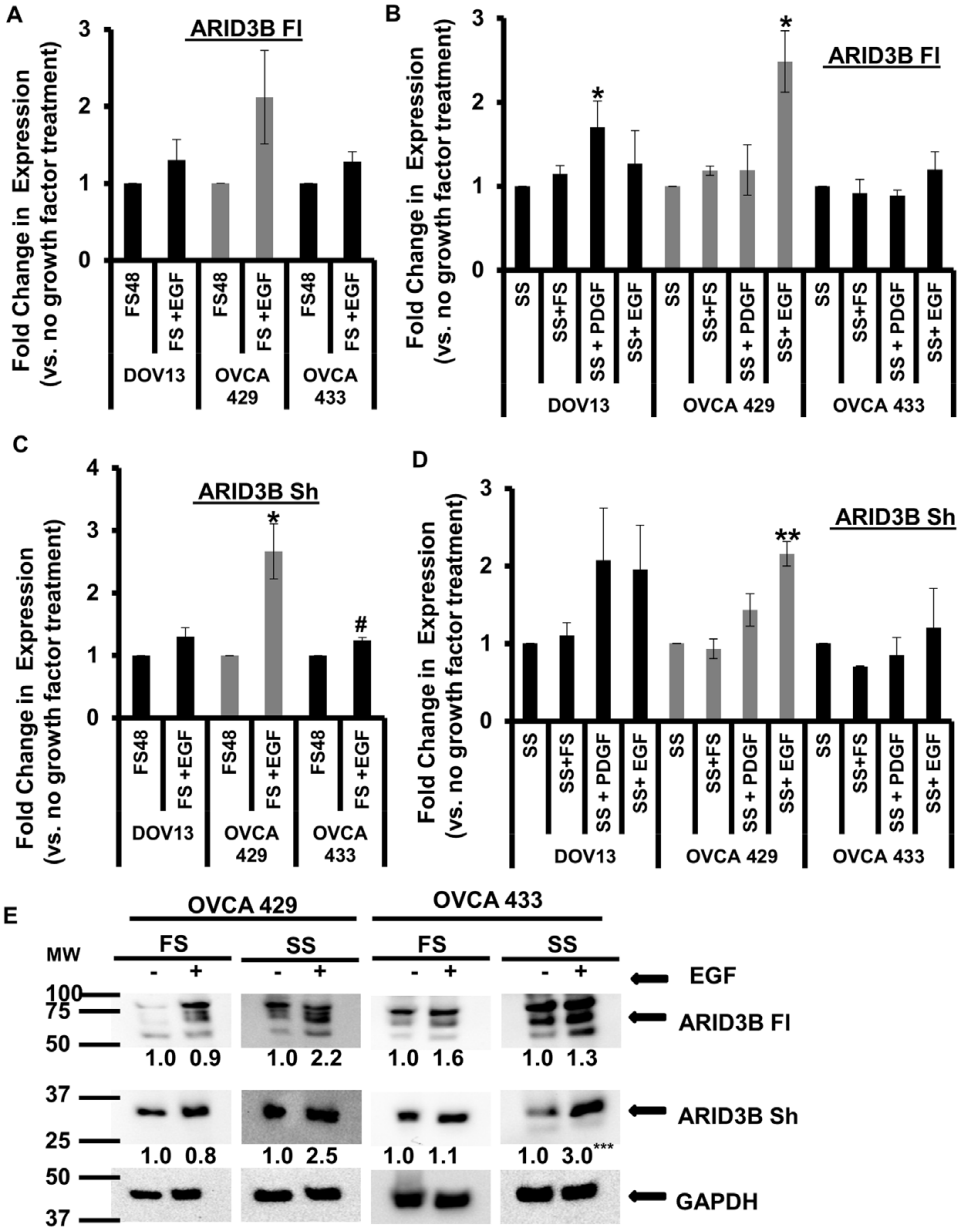
EGFR modestly regulates the expression of ARID3B splice forms. QRT-PCR for ARID3B Fl and ARID3B Sh was performed on total RNA isolated from OVCA 429, OVCA 433 and DOV13 ovarian cancer cells grown in 10% serum conditions (FS) (**A and C**) or serum starved (SS) (**B and D**) for 24 h and treated with or without 20 nM of EGF or 10 nM of PDGF for an additional 24 h. QRT-PCR was performed for ARID3B Fl (**A and B**) and ARID3B Sh (**C and D**). Expression was normalized to 18 s rRNA. Fold expression was normalized to non-EGF treated cells (SS or FS) and was expressed as the mean ± SEM of triplicate measurements. Statistical analysis was performed to determine if there were any significant changes between FS vs. treatment with FS and EGF in all three ovarian cancer cell lines and for both ARID3B isoforms (**A and C**). We also performed statistical analysis to determine if there were any significant changes between SS vs. treatment with SS and FS or PDGF or EGF in all three ovarian cancer cell lines and for both ARID3B isoforms (**B and D**). **E**. OVCA 429 and OVCA 433 ovarian cancer cells grown in serum starved (SS) or 10% serum conditions (FS) for 24 h and treated with or without 20 nM of EGF for an additional 24 h. Whole cell lysates were analyzed by western blot using anti-ARID3B and anti-GAPDH (control) antibodies. Results were normalized to GADPH expression and compared to the expression of cell not treated with EGF (SS or FS). The densitometry evaluation of the western blot analyses for ARID3B Fl and ARID3B Sh in ovarian cancer cells were analyzed (value under blot). Statistical analysis was performed to determine if there were any significant changes between (FS) vs. (FS and EGF) and (SS) vs. (SS and EGF) in all three ovarian cancer cell lines and for both ARID3B isoforms fold expression was expressed as the mean of triplicate measurements. [*p<0.05, **p<0.005, ***p<0.0005 and ^#^p<0.01].

Tumor cells often have enhanced growth factor signaling [22]. Since we found that two of the ovarian cancer (OVCA 433 and OVCA 429) cell lines exhibited high levels of mRNA expression of ARID3B Sh, we wanted to investigate whether ARID3B mRNA levels are regulated in these cell lines by growth factor signaling. Both EGFR and ARID3B are overexpressed in ovarian cancer [2], [18]. To assess if the expression of ARID3B splice forms is regulated by growth factor signaling (both EGF and platelet derived growth factor (PDGF)), we grew DOV13, OVCA 429 and OVCA 433 ovarian cancer cells under serum starved (SS) or 10%/full serum (FS) conditions for 24 h and treated them with or without 20 nM EGF or 10 nM PDGF for an additional 24 h and analyzed the mRNA expression of ARID3B splice forms by QRT-PCR using 18 S rRNA as a control (Fig. 3A, 3B, 3C and 3D). The mRNA expression of ARID3B Fl and ARID3B Sh increased in OVCA 429 cells treated with either EGF in the presence or absence of serum. OVCA 429 cells are more responsive to EGF than both OVCA 433 and DOV13 cells with respect to invasive properties and regulation of proteases [23] (data not shown). EGF treatment in the absence of serum induced mRNA expression of ARID3B Fl and ARID3B Sh in OVCA 429 cells (2.5-fold (p<0.05) and 2.2-fold (p<0.005), respectively), but not in OVCA 433 cells (Fig. 3B and 3D). Western blot analysis of OVCA 433 and OVCA 429, demonstrated that treatment with EGF appears to have a modest increase ARID3B Fl and ARID3B Sh protein, although the densitometry analysis suggests that this trend is not significant (Fig. 3E). These findings demonstrate that EGFR signaling leads to a mild increase in ARID3B Fl and ARID3B Sh mRNA but had little impact on protein.

### EGF Induces PEA3 Binding to the ARID3B Promoter

**Figure 4:**
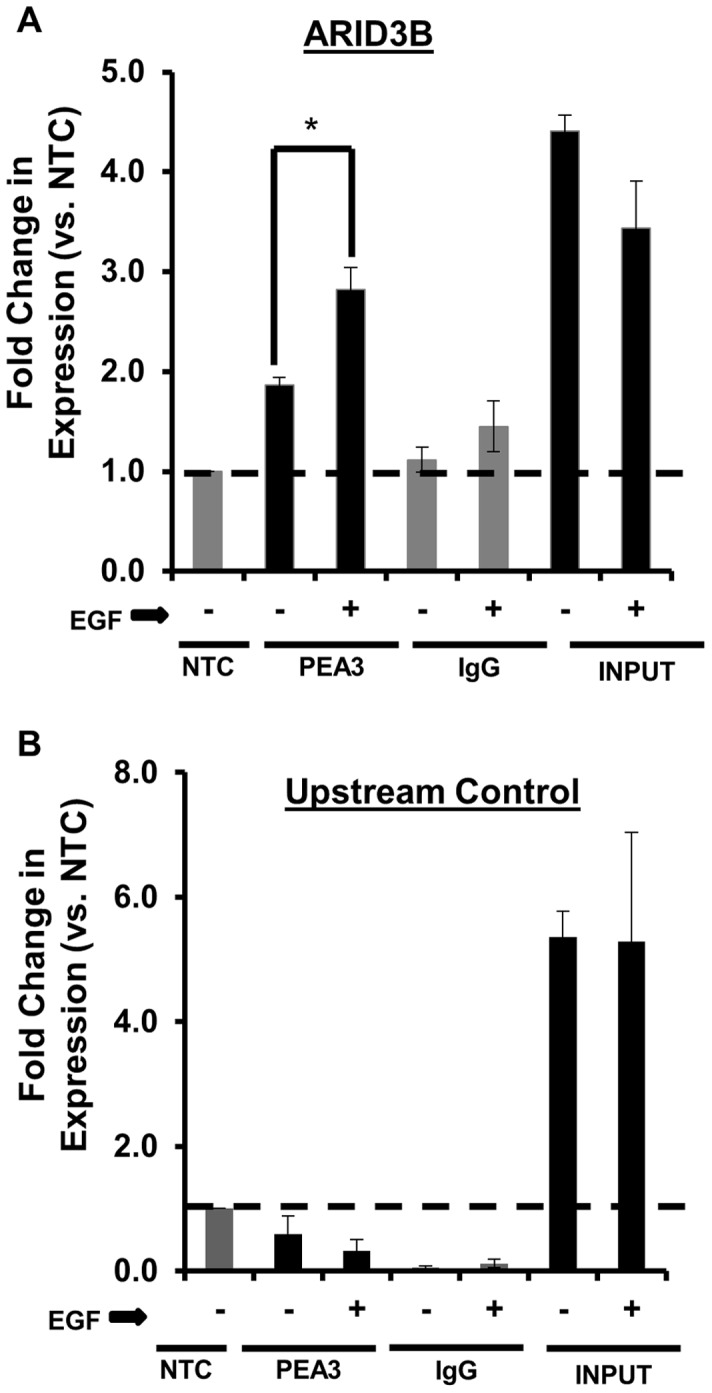
EGFR regulates PEA3 binding to the ARID3B promoter. Chromatin immunoprecipitation (ChIP) analysis confirmed the binding PEA3 to the ARID3B promoter region. ChIP was performed on OVCA 429 cells serum starved (SS) for 24 h then treated with or without 20 nM EGF for 24 h. Quantitative PCR analysis was conducted for (**A**) a region of the ARID3B promoter containing ETS/PEA3 binding site and (**B**) a promoter sequence upstream of the ETS binding site. ChIP was performed using IgG (negative control) or anti-PEA3. Expression was normalized to NTC (no template control). The no template control (NTC) expression was a boundary limit (expression fold  = 1), any fold-expression below this limit (expression <1) was considered to be negligible. Statistical analysis was performed to determine if there were any significant changes in chromatin binding between cells without EGF vs. cells treated with EGF. Fold expression was expressed as the mean ± SEM of triplicate measurements. [*p<0.05].

The increase in ARID3B mRNA in response to EGFR signaling suggests that ARID3B is transcriptionally activated as a result of growth factor induced signal transduction. Previously we demonstrated that the ETS family transcription factor PEA3 is regulated by EGFR in ovarian cancer cells and critical to EGFR induced invasion [2], [20]. To examine whether EGFR signaling induces PEA3 association with the ARID3B promoter, we serum starved OVCA 429 cells for 24 h, treated the cells with or without 20 nM EGF for an additional 24 h and performed chromatin immunoprecipitation (ChIP) to assess the binding of PEA3 to ARID3B promoter. Cross-linked chromatin was immunoprecipitated with anti-PEA3 or IgG antibody and DNA was amplified by quantitative PCR with primers specific for a portion of the ARID3B promoter containing ETS binding site. In the ARID3B promoter, there is an ETS site (GAGGAA) located 822 bp upstream of transcription start. There are also additional ETS sites between 4000 bp-822 bp upstream of transcription start. We chose to focus on the ETS site closest to the ATG. As controls we used an upstream region of the ARID3B promoter that lacks ETS sites (negative control). Using the no template control (NTC) expression as a boundary limit (expression fold  = 1), any fold-expression below this limit (expression <1) was considered to be negligible. Our findings demonstrate that treatment of cells with 20 nM EGF enhances PEA3 association with the ETS site found in the ARID3B promoter (Fig. 4). PEA3 binding to the ARID3B promoter after treatment with EGF significantly increased by 1.5-fold (p<0.05), in the PEA3 immunoprecipitated samples (Fig. 4A). As controls, for the input samples we show the amplification of the DNA surrounding the ETS site in the ARID3B promoter and the upstream region of the ARID3B promoter (Fig. 4A and 4B). The data demonstrates that PEA3 directly binds to the ARID3B promoter in response to EGFR activation.

### ARID3B Isoforms are Located in Different Subcellular Regions

**Figure 5:**
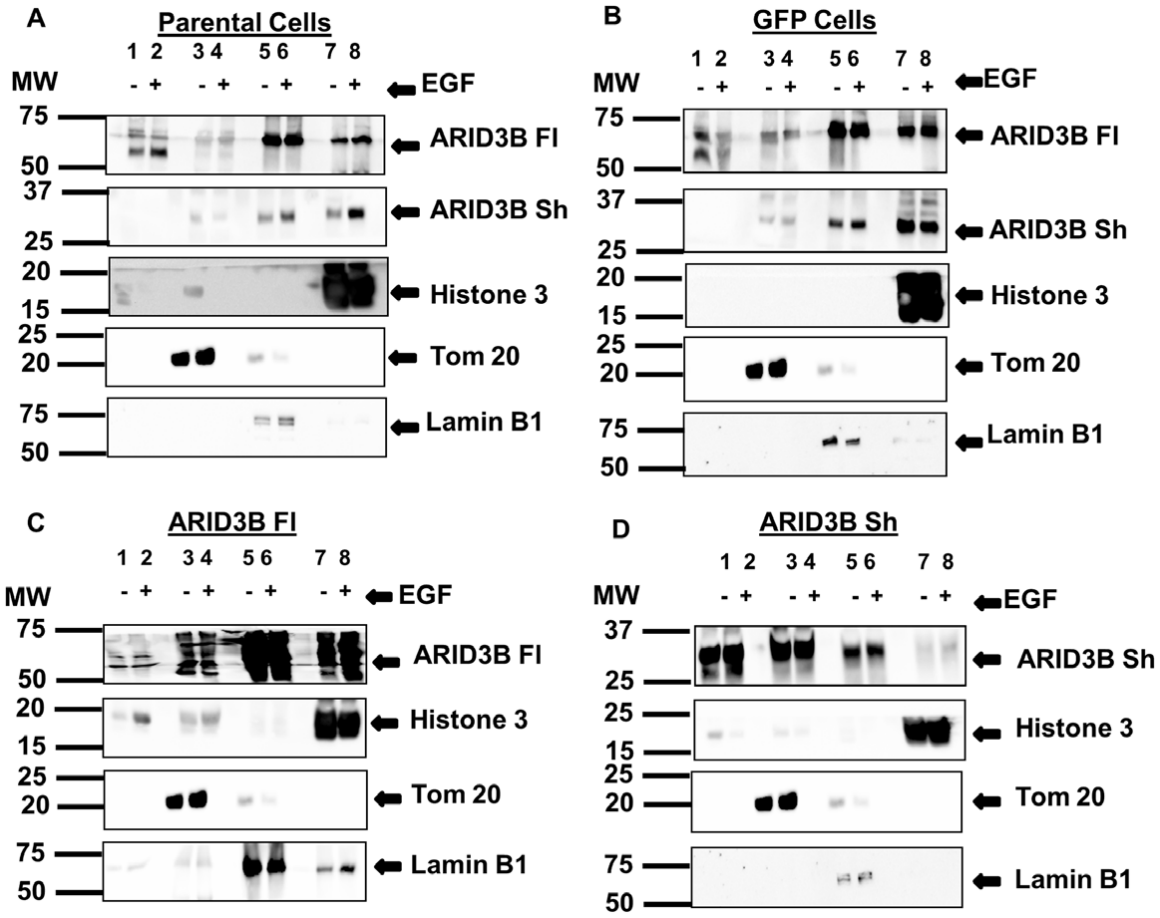
Subcellular localization of ARID3B splice forms. Parental OVCA 429 cells (**A**) or OVCA 429 cells transduced with GFP (**B**), ARID3B Fl (**C**) or ARID3B Sh (**D**) were serum starved (SS) for 24 h, treated with or without 20 nM EGF for 24 h, and fractionated into cytoplasmic, membrane, nuclear and chromatin-bound extract fractions. Western blot was performed for ARID3B, Tom 20, Histone H3 and Lamin B1. *Cytoplasmic fraction* [lanes 1 (no EGF) and 2 (20 nM EGF)], *membrane fraction* [lanes 3 (no EGF) and 4 (20 nM EGF)], *nuclear soluble fraction* [lanes 5 (no EGF) and 6 (20 nM EGF)] and *chromatin-bound fraction* [lanes 7 (no EGF) and 8 (20 nM EGF)].

To investigate the cellular localization of ARID3B Fl and ARID3B Sh in ovarian cancer cells, we fractionated cells using the Pierce Subcellular Fractionation Kit (Fig. 5). We transduced human ovarian cancer cells, OVCA 429, with lentiviral particles expressing GFP, ARID3B Fl or ARID3B Sh, and treated cells with or without 20 nM EGF. Transduced cells and the parental cell lines were separated into cytoplasmic, membrane, soluble nuclear and chromatin-bound nuclear fractions. In parental OVCA 429 and GFP transduced OVCA 429 cells, endogenous ARID3B Fl is found in all cellular fractions but is predominately located in the nuclear soluble and chromatin-bound nuclear fractions (Fig. 5A and 5B). When ARID3B Fl was overexpressed it was observed mainly in the nuclear soluble and chromatin-bound nuclear fractions (Fig. 5C, Figure S3A). Endogenous ARID3B Sh was present in the nuclear fraction as well as the chromatin-bound nuclear fraction (Fig. 5A and 5B). In contrast when ARID3B Sh was overexpressed we observed an accumulation in the cytosol and mitochondria/membrane fraction (Fig. 5D, Figure S3B). EGF treatment for 24 h on parental cell lines and transduced cells did not alter the localization of ARID3B isoforms. Additionally, Lamin B1 (nuclear marker), Histone H3 (chromatin marker) and Tom 20 (mitochondria marker was used as a marker for the membrane fraction which contains all membrane bound organelles), were used to indicate efficient separation. Similar results were obtained when SKOV3 and OVCA 433 cells were transduced with GFP, ARID3B Fl, or ARID3B Sh and then fractionated (data not shown).

**Figure 6:**
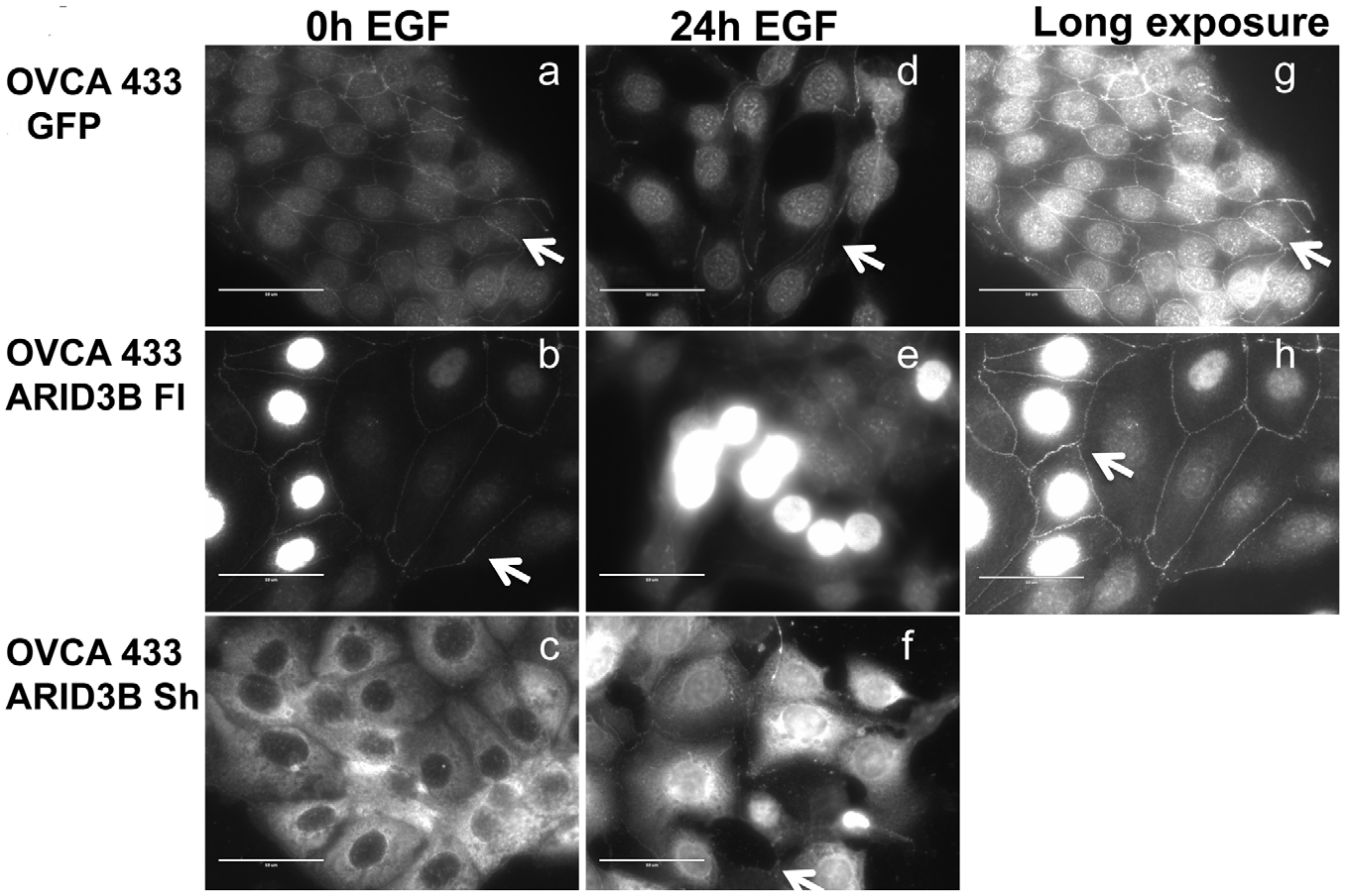
Localization of ARID3B splice forms by immunofluorescence. Immunofluorescence on OVCA 433 cells expressing GFP (**a, d, g**), ARID3B Fl (**b, e, h**), and ARID3B Sh (**c, f**). Cells are untreated (0 h EGF) (**a, b, c**) or treated with EGF for 24 h (**d, e, f**). Arrow indicates plasma membrane staining. Scale bars = 50 um. Panels g and h are long exposures of panels **a and b** in order to better visualize membrane associated ARID3B. Scale bar  = 50 uM.

To further examine ARID3B subcellular localization we employed immunofluorescence analysis. For this investigation we serum-starved OVCA 433 (not shown), OVCA 433 GFP, OVCA 433 ARID3B Fl, and OVCA 433 ARID3B Sh cells for 24 h and then either left cells untreated or treated cells for 24 h with 20 nM EGF. The same experiment was performed on OVCA 429 and SKOV3 cells and yielded similar results (data not shown). We then performed immunofluorescence for ARID3B using an antibody that recognizes both ARID3B Fl and ARID3B Sh. In untreated OVCA 433 GFP cells, immunofluorescence was observed predominantly in the nucleus with some ARID3B localized to the plasma membrane (Fig. 6a and g). EGF treatment does not affect the localization of endogenous ARID3B Fl (Fig. 6d). OVCA 433 ARID3B Fl cells exhibit strong nuclear staining for ARID3B that is significantly increased by EGF treatment (p<0.01) (Fig. 6b, h and e). Using Image J we generated circles around 75 nuclei in ARID3B Fl transduced cells that were untreated and 75 nuclei from EGF treated cells. Image J reported the mean fluorescence intensity of each nucleus. The average intensity of ARID3B Fl expression in untreated cells was 40 (arbitrary units) versus 55 (arbitrary units) in EGF treated ARID3B Fl overexpressing cells (p<0.01). We then examined OVCA 433 cells that overexpress ARID3B Sh. OVCA 433 ARID3B Sh cells exhibit strong cytoplasmic staining for ARID3B (Fig. 6c). Upon EGF treatment we detect increased nuclear ARID3B in cells overexpressing ARID3B Sh (Fig. 6f). Dual immunofluorescence with Mitotracker (Invitrogen) and ARID3B and isolation of mitochondria from ARID3B Sh overexpressing OVCA 433 and OVCA 429 cells reveal that a small amount of exogenous ARID3B is mitochondrial (data not shown).

### ARID3B Fl but not ARID3B Sh Induces Apoptosis

**Figure 7:**
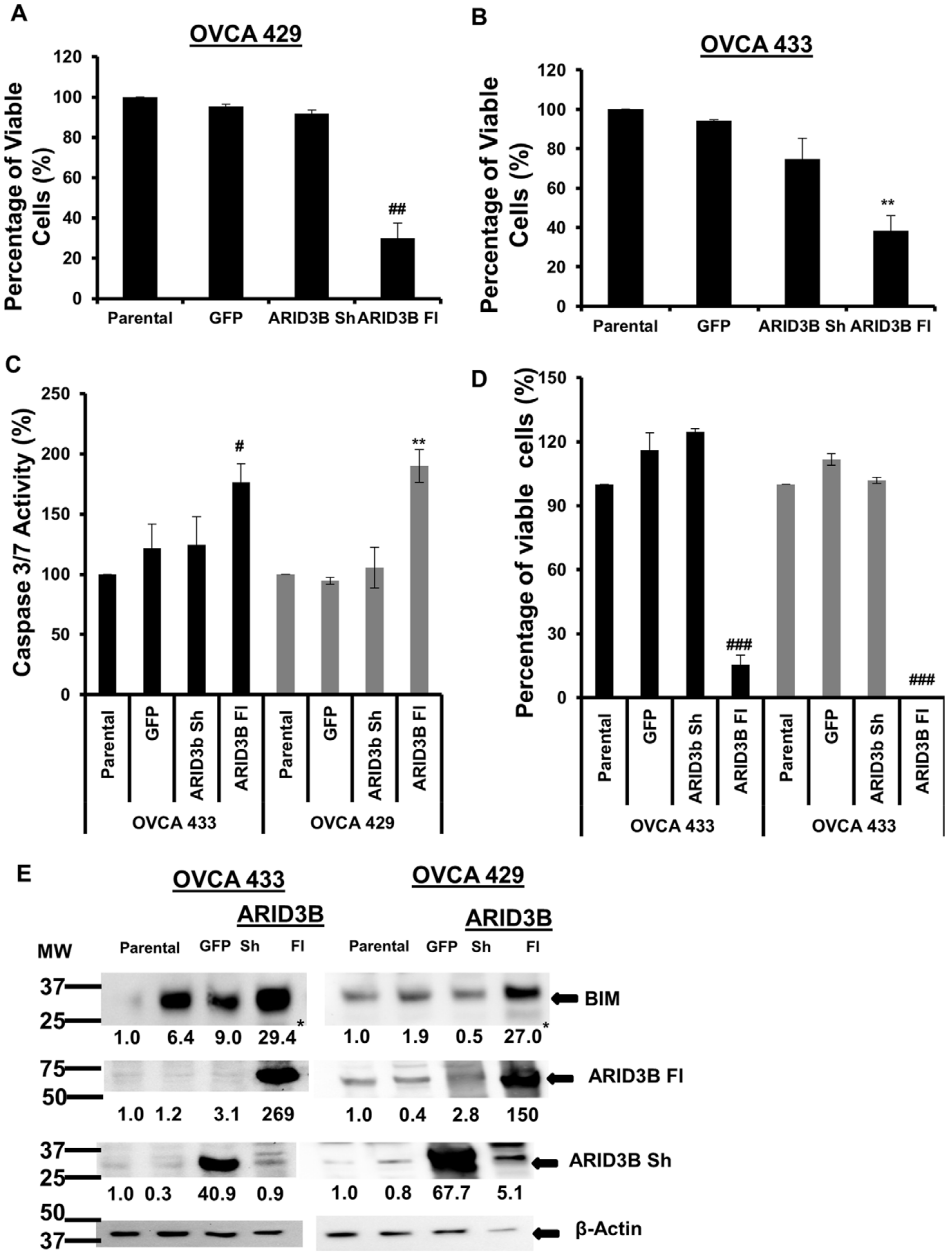
ARID3B Fl induces apoptosis in ovarian carcinoma cells. Flow cytometry analysis using Annexin V and 7-AAD was used to evaluate the apoptosis in OVCA 429 (**A**) and OVCA 433 (**B**) overexpressing the ARID3B splice forms. OVCA 429 and OVCA 433 cells were lentivirally transduced with ARID3B Fl, ARID3B Sh and GFP (control). Flow cytometry for Annexin V and 7-AAD was performed on OVCA 429 (A) and OVCA 433 (B) the percentage of viable cells was expressed as the mean ± SEM of triplicate measurements. **C**. The Caspase-Glo 3/7 assay for Caspase 3/7 activities was performed on OVCA 433 and OVCA 429 ovarian cancer cells 72 h post transduction. Caspase-Glo 3/7 activity is shown in reference to parental cell and normalized to the cell count after transduction with GFP, ARID3B Sh and ARID3B Fl for 72 h. Caspase activity of parental cells was defined as 100%. Caspase activity was expressed as the mean ± SEM of triplicate measurements. **D**. Cell viability analysis using MTT assay was used to confirm the apoptotic phenotype of ARID3B Fl. MTT assay was performed on OVCA 429 and OVCA 433 transduced with ARID3B Fl, ARID3B Sh and GFP cells for 72 h and the percentages of viable cells (compared to parental cells) were expressed as the mean ± SEM of triplicate measurements. **E**. Western blot using anti-ARID3B, anti-BIM and anti- β-actin (control) antibodies on transduced OVCA 433 and OVCA 429 cells. The densitometry evaluation of the western blot analyses for BIM was analyzed (value under blot). Results were normalized to β-actin expression and compared to parental ovarian cancer cells Statistical analysis was performed to determine if there were any significant changes between parental ovarian cancer cells vs. GFP, ARID3B Sh or ARID3B Fl transduced ovarian cancer cells. [**p<0.005, ***p<0.0005, ^#^p<0.01,^##^p<0.001 and ^###^p<0.0001].

After examining the expression and localization of ARID3B isoforms, we wanted to determine the functions of these gene products in epithelial ovarian cancer cells. To evaluate the functional characteristics of the two ARID3B splice forms, OVCA 433 and OVCA 429 cells were transduced with lentivirus containing GFP (control), ARID3B Sh or ARID3B Fl. We observed that cells transduced with ARID3B Fl underwent cell death within a few days of transduction. To assess if ARID3B isoforms induce apoptosis we performed Annexin V analysis. In brief, OVCA 433 and OVCA 429 cells were transduced with GFP, ARID3B Fl or ARID3B Sh for 72 hours and cells were harvested and stained with both Annexin V and 7-AAD. Analysis was performed via flow cytometry. Seventy percent of OVCA 429 cells (p<0.0005) (Fig. 7A) and 62% of OVCA 433 cells (p<0.0005) (Fig. 7B) overexpressing ARID3B Fl were undergoing apoptosis after 72 hours. In contrast, there was no significant difference in the percentage of live GFP expressing cells after 72 h of transduction. An average of 95% of GFP transduced OVCA 433 and OVCA 429 cell were viable after transduction. Eighty-nine percent of ARID3B Sh expressing cells OVCA 433 and OVCA 429 cell were viable after 72 hours of viral transduction (Fig. 7A and 7B). To verify this finding, Caspase 3 and Caspase 7 enzymatic activities were evaluated. OVCA 433 and OVCA 429 cells were transduced with GFP, ARID3B Fl or ARID3B Sh. Seventy-two hours post transduction we performed Caspase-Glo 3/7 assay to evaluate the enzymatic activity of Caspase 3 and 7. Caspase 3 and Caspase 7 enzymatic activity increased significantly by 1.8-fold (OVCA 433; p<0.01) and 1.9-fold (OVCA 429; p<0.005) in cells treated with ARID3B Fl relative to parental cells (Fig. 7C). In addition, 3-(4,5-Dimethylthiazol-2-yl)-2,5-diphenyltetrazolium bromide (MTT) assay was performed under the same conditions as the Caspase-Glo 3/7 assay. MTT assay indicated significant decreases in cell viability of 6.5- fold (OVCA 433; p<0.001) and 200-fold (OVCA 429; p<0.001) for ARID3B Fl transduced cells at 72 h post transduction relative to the parental cells (Fig. 7D). This decrease was not observed with GFP or ARID3B Sh transduced cells. To confirm that ARID3B Fl leads to the induction of apoptotic pathways, we performed immunoblot analysis for endogenous BIM (Bcl-2 interacting mediator of cell death). BIM is a pro-apoptotic member of the Bcl-2 family [24], [25]. BIM directly binds to the anti-apoptotic Mcl-1 and Bcl-2 proteins countering their pro-survival effects [25]. BIM also interacts directly with BAX [26]. After 72 hours of ARID3B Fl transduction in OVCA 433 and OVCA 429 cells, BIM expression is increased (Fig. 7E). We demonstrated that the expression level of BIM increased 29.4-fold in OVCA 433 (p<0.005) and 27.0-fold in OVCA 429 (p<0.005) relative to the parental cells. Our data therefore indicates that ARID3B Fl not ARID3B Sh acts as a pro-apoptotic factor in ovarian cancer cells.

### Overexpression of ARID3B Fl Induces the Expression of Genes Involved in the Tumor Necrosis Factor Alpha **(**TNFα) Apoptotic Pathway

To identify genes involved in ARID3B Fl-induced apoptosis a quantitative PCR based gene array was performed (n = 1) (TaqMan Human Apoptosis Array: detecting 92 human apoptotic related genes). Apoptotic inducing genes were identified by comparing mRNA from ARID3B Sh transduced OVCA 429 cells with ARID3B Fl transduced OVCA 429 cells (Table S1). Among the 92 apoptosis- related genes tested, several genes involved in the induction of apoptosis increased. Noticeably, expression of the following genes were increased in ARID3B Fl transduced OVCA 429 cells compared to ARID3B Sh transduced OVCA 429 cells TRAIL, TNF-R2, TNFα, TRADD, BID and BAK, indicating a correlation between ARID3B Fl overexpression with several pro-apoptotic genes. We also observed a decrease in expression of several pro-survival genes. XIAP, Bcl-2 and Bcl-xL expression decreased in ARID3B Fl transduced OVCA 429 cells compared to ARID3B Sh transduced OVCA 429 cells, indicating a negative correlation between ARID3B Fl overexpression with several pro-survival genes. This initial finding indicates that the overexpression of ARID3B Fl induces the expression of several pro-apoptotic genes and inhibits several pro-survival genes. Importantly, we identified the TNFα/TRAIL signaling pathways as potential mediators of ARID3B Fl-induced apoptosis.

To validate the observation in the gene array data (Table S1 (n = 1)), the mRNA expression of nine gene genes (BCL10, BID, Caspase 7, Caspase 10, Caspase 8, TNFα, TRADD, TNF-R2 and TRAIL) and the ARID3B isoforms was confirmed in OVCA 429 and OVCA 433 transduced with lentivirus containing GFP (control), ARID3B Sh or ARID3B Fl. ARID3B Fl overexpression in ovarian cancer cell lines significantly increased the expression of all nine apoptotic related genes, while ARID3B Sh overexpression failed to do so (Table 1 and 2). This finding supports a correlation between expression of ARID3B Fl and the induction of apoptosis. Expression of TNFα, TRADD, TNF-R2 and TRAIL were significantly increased by 19.2-, 2.6-, 59.6- and 3.0-fold respectively in OVCA 429 cells (Table 2) and 20.0-, 1.9-, 2.7- and 2.0-fold respectively in OVCA 433 cells (Table 1). Caspase 7 and Caspase 10 were increased by 2.0- and 2.9-fold respectively in OVCA 429 cells (Table 2) and 2.0- and 2.0-fold respectively in OVCA 433 cells (Table 1).

**Table 1:** Identification of ARID3B Fl-induced pro-apoptotic genes in OVCA 433 cells.

	Fold Expression (Mean ±SEM)			
Gene Target	Parental cell	GFP	ARID3B Sh	ARID3B Fl
ARID3B Sh	1.0±0.00	0.5±0.20	1907.1±434	5.2±1.10
ARID3B Fl	1.0±0.00	0.6±0.24	1.2±0.17	321.6±9.47
BCL10	1.0±0.00	0.5±0.03	1.1±0.01	1.4±0.04^##^
BID	1.0±0.00	0.5±0.02	1.1±0.09	1.6±0.09**
CASP7	1.0±0.00	0.4±0.01	0.9±0.07	2.0±0.014**
CASP10	1.0±0.00	0.4±0.06	0.9±005	2.0±0.00^###^
CASP8	1.0±0.00	0.5±0.07	0.9±0.07	1.3±0.06^#^
TNFα	1.0±0.00	0.3±0.02	0.9±0.05	20.00±1.55***
TRADD	1.0±0.00	0.3±0.00	1.0±0.23	1.87±0.09^##^
TNF-R2	1.0±0.00	0.3±0.02	1.0±0.02	2.7±0.25**
TRAIL	1.0±0.00	0.2±0.02	0.5±0.06	2.0±0.19^#^

QRT-PCR analysis of potential gene targets (Table S1) of OVCA 433 cells transduced with GFP, ARID3B Sh and ARID3B Fl. Statistical analysis for each target gene was performed by comparing the transduced cells mRNA expression to the corresponding Parental cell mRNA expression. **p<0.005, ***p<0.0005, ^#^p<0.01, ^##^p<0.001, ^###^p<0.0001.

**Table 2:** Identification of ARID3B Fl-induced pro-apoptotic genes in OVCA 429 cells.

	Fold Expression (Mean ±SEM)			
Gene Target	Parental cell	GFP	ARID3B Sh	ARID3B Fl
ARID3B Sh	1.0±0.00	1.1±0.30	3522.8±259.00	4.8±0.20
ARID3B Fl	1.0±0.00	0.9±0.21	1.1±0.05	395.9±16.84
BCL10	1.0±0.00	0.8±0.06	0.9±0.03	1.2±0.09*
BID	1.0±0.00	0.9±0.02	1.1±0.09	3.0±.67*
CASP7	1.0±0.00	0.9±0.019	1.0±0.01	2.0±0.14**
CASP10	1.0±0.00	0.8±0.20	1.1±0.03	2.9±0.33**
CASP8	1.0±0.00	0.9±0.15	1.0±0.05	1.3±0.04**
TNFα	1.0±0.00	0.8±0.16	0.4±0.04	19.2±5.00*
TRADD	1.0±0.00	0.7±0.16	1.1±0.09	2.62±0.41*
TNF-R2	1.0±0.00	0.7±0.09	1.2±0.06	59.6±18.89*
TRAIL	1.0±0.00	0.9±0.05	1.0±0.27	3.0±0.45*

QRT-PCR analysis of potential gene targets (Table S1) of OVCA 433 cells transduced with GFP, ARID3B Sh and ARID3B Fl. Statistical analysis for each target gene was performed by comparing the transduced cells mRNA expression to the corresponding Parental cell mRNA expression. *p<0.05, **p<0.005.

**Figure 8:**
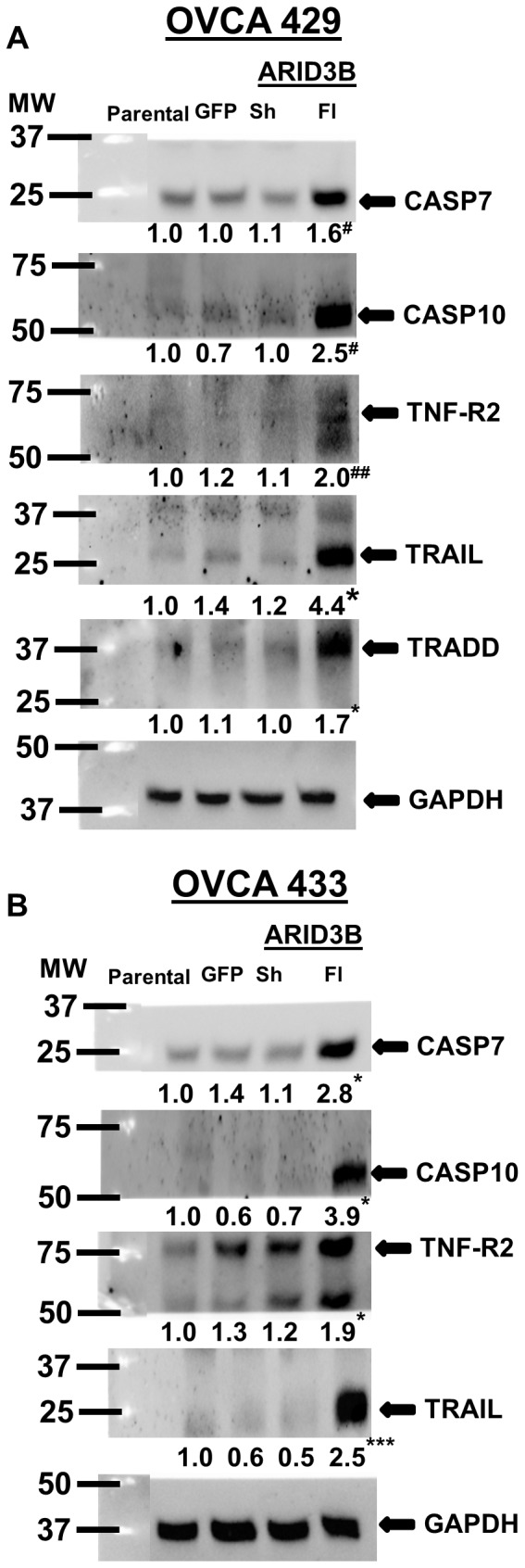
ARID3B Fl induces apoptosis in ovarian carcinoma cells via TNFα/TRAIL pathways. Western blot using anti-Caspase 7, anti-Caspase 10, anti-TNF-R2, anti-TRAIL, anti-TRADD and anti- GAPDH (control) antibodies on transduced OVCA 429 (**A**) and OVCA 433 (**B**) cells. The densitometry evaluations of the western blots were analyzed (value under blot). Results were normalized to GAPDH expression and compared to parental ovarian cancer cells. Statistical analysis was performed to determine if there were any significant changes between parental ovarian cancer cells vs. GFP, ARID3B Sh or ARID3B Fl transduced ovarian cancer cells. [*p<0.05, ***p<0.0005, ^#^p<0.01 and ^##^p<0.001].

To confirm that ARID3B Fl leads to the induction of apoptosis via the TNFα/TRAIL apoptotic pathways we performed immunoblot analysis for the following five protein targets; Caspase 7, Caspase 10, TNF-R2, TRAIL and TRADD in OVCA 429 and OVCA 433 cells transduced with lentivirus containing GFP (control), ARID3B Sh or ARID3B Fl. ARID3B Fl overexpression in both ovarian cancer cell lines significantly increased the expression of the above listed proteins, while ARID3B Sh overexpression did not (Fig. 8). Expression of Caspase 7, Caspase 10, TNF-R2, TRAIL and TRADD were significantly increased by 1.6-, 2.5-, 2.0- and 1.7-fold respectively in OVCA 429 cells (Fig. 8A) and 2.8-, 3.9- and 2.5- fold respectively in OVCA 433 cells (Fig. 8B).

**Figure 9:**
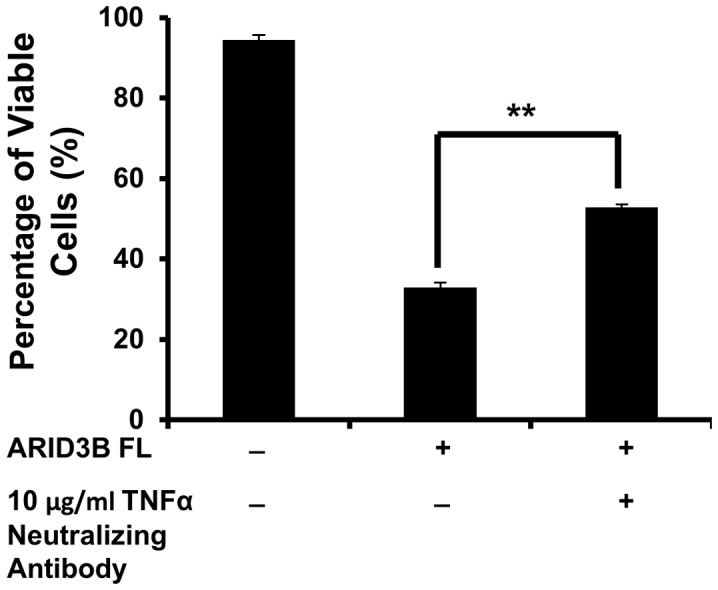
Anti-TNFα neutralizing antibody impairs ARID3B Fl apoptotic activity. Flow cytometry analysis using Annexin V and 7-AAD was used to evaluate the apoptosis in OVCA 429 overexpressing the ARID3B Fl treated with or without 10 µg/ml anti-TNFα-neutralizing antibody. Twenty four hours pre-transduction with ARID3B Fl, OVCA 429 cells were treated with or without 10 µg/ml of human an anti-TNFα neutralizing antibody. Seventy two hours post transduction flow cytometry for Annexin V and 7-AAD was performed on OVCA 429 cells and the percentage of viable cells was expressed as the mean ± SEM of triplicate measurements. Statistical analysis was performed to determine if there were any significant changes between ARID3B Fl transduced cells vs. anti-TNFα treated ARID3B Fl transduced cells. [**p<0.005].

Furthermore we performed TNFα immunoassay to analyze the effects of ARID3B isoforms on TNFα secretion in the cells transduced with lentivirus containing GFP (control), ARID3B Sh or ARID3B Fl. The TNFα protein concentrations were below the minimum detectable concentration of the assay (1.6 pg/mL) in both OVCA 433 and OVCA 429 parental cells and cells transduced with GFP or ARID3B Sh (Table 3). However, we observed a significant expression of TNFα protein levels in cell culture supernates; 38.1 pg/mL (p<0.0005) and 12.1 pg/mL (p<0.0001) of TNFα protein respectively in OVCA 433 and OVCA 429 cells transduced with ARID3B Fl (Table 3). To further investigate whether the TNFα pathway contributes to ARID3B Fl-induced apoptosis, we treated cells with a TNFα neutralizing antibody to impair the TNF pathway (Fig. 9). OVCA 429 cells were treated with or without 10 µg/ml TNFα neutralizing antibody 24 h prior to being transduced with ARID3B Fl lentiviral particles. To assess if TNFα neutralizing antibody impaired ARID3B Fl-induce apoptosis, we performed Annexin V analysis as described above after 72 h post transduction. TNFα neutralizing antibody treatment of ARID3B Fl OVCA 429 cells significantly increased the levels of viable cells by 1.6-fold (p<0.005) compared to untreated ARID3B Fl OVCA 429 cells. Therefore, ARID3B Fl in part induced apoptosis via TNFα signaling.

**Table 3:** TNFα Concentration of Cell Culture Supernate.

	Protein Concentration (pg/ml) (Mean ±SEM)			
Cell line	Parental cell	GFP	ARID3B Sh	ARID3B Fl
OVCA 429	na	na	na	12.1±1.17***
OVCA 433	na	na	na	38.1±1.59^###^

TNFα immunoassay analysis of OVCA 429 and OVCA 433 cells transduced with GFP, ARID3B Sh and ARID3B Fl. Statistical analysis was performed by comparing the transduced cells TNFα protein expression to the corresponding parental cell TNFα protein expression. ***p<0.0005, ^###^p<0.0001.na indicates protein levels below the minimum detectable dose of 1.6 pg/mL.

## Discussion

Ovarian cancer progression results from activation of multiple pathways involving both genetic and epigenetic changes. However, the mechanisms that contribute to ovarian cancer progression are poorly understood. We previously demonstrated that ARID3B is overexpressed in human serous ovarian cancer suggesting that it may play a role in the tumor progression. Upon further investigation we discovered that ARID3B is alternatively spliced in ovarian cancer cells. This splicing yields a 28 kDa isoform, ARID3B Sh, which is highly expressed in several cancer cell lines. The presence of this alternate splice form suggests that multiple splice forms of ARID3B may play a role in the progression of ovarian cancer.

Upon determining that ARID3B isoforms were both expressed highly in ovarian cancer cells we wanted to assess if they were regulated by EGFR. EGFR is not only associated with poor prognosis in ovarian cancer it also promotes alternative splicing of Caspase-9 in favor of Caspase-9b [17], [18], [21]. Since we previously identified ARID3B as a gene target of the EGFR-responsive miR-125a [2], we postulated that ARID3B expression or splicing may be regulated by EGF signaling. However, EGF treatment did not substantially regulate the expression of both ARID3B splice forms. To determine if ARID3B is directly regulated by PEA3 in response to EGFR activation, we performed ChIP for PEA3 binding in response to EGF. In response to EGF treatment PEA3 bound to the ARID3B promoter. However since there is not a sizable increase in ARID3B mRNA, other cofactors or signaling events may be required for ARID3B transcriptional activation. Other oncogenes such as MYCN may more strongly induce ARID3B in human cancer [14], [27].

One of the clear goals of the study is to identify the function of the ARID3B splice forms. Understanding the function of the ARID3B splice forms may lead to a better understanding of the progression of ovarian cancer. In this study we demonstrated that ARID3B Fl, but not ARID3B Sh induces apoptosis in ovarian cancer cell lines (Fig. 7). Furthermore we show that ARID3B induces expression of TNFα, TRAIL, TNF-R2, and TRADD, which are involved in TNFα-mediated signaling. Inhibition of TNFα activity using a neutralizing antibody prior to transduction with ARID3B Fl confirmed that ARID3B induction of TNFα signaling is one mechanism by which ARID3B Fl induces apoptosis. TNFα and the related protein TRAIL regulate apoptosis via their death receptors [28], [29]. TNFα promotes both cell survival and cell death depending on the receptors that they bind and the signaling complexes that are formed in response to ligand binding [30], [31], [32], [33], [34]. TRAIL has been suggested to be a promising therapeutic for cancer since many cancer cell lines, but not normal cells undergo apoptosis upon treatment with TRAIL [28], [35], [36]. The therapeutic value of TRAIL is complicated by TRAIL resistance in many cell lines [28], [37], [38]. Therefore the identification of factors that regulate TNFα and TRAIL may be therapeutically important. We show that ARID3B Fl induces the mRNA expression of several factors involved in TNFα/TRAIL signaling. ARID3B Fl also induces other pro-apoptotic genes such as Caspase10, and Caspase 7, and BIM. Of note, we also found that ARID3B Fl may inhibit the expression of the pro-survival genes Bcl-2, Bcl-xL and XIAP. Several of the proteins assayed by mRNA expression are regulated by post-translational modifications (Bad), proteolysis (Bid, Caspase 7 and Caspase 10), or through protein: protein interactions (Bak) and thus small changes in mRNA expression may not alter function. Using western blot analysis we demonstrated that ARID3B Fl expression leads to increased expression of pro-apoptotic proteins in addition to mRNA. Additionally, the activity of Caspases 3/7 is elevated, suggesting that the increased in expression of the apoptotic proteins leads to cell death. These results suggest that overexpressing ARID3B Fl in ovarian tumors may lead to tumor cell death.

ARID3B Fl and ARID3B Sh display different patterns of subcellular localization. ARID3B Fl is predominately nuclear however a fraction of ARID3B Fl is localized to the plasma membrane. Our data are interesting in light of the previous studies on ARID3B localization and the subcellular localization of ARID3A and ARID3C [7]. It was previously reported that endogenous ARID3B is exclusively expressed in the nucleus in B-cell lines and exogenous ARID3B is exclusively nuclear in Cos7 cells [10]. We provide compelling evidence that in epithelial ovarian cancer cells that ARID3B is not exclusive to the nucleus. Clearly ARID3B Fl is predominantly nuclear, but some of ARID3B Fl is associated with the plasma membrane and present in the cytosol. We predict that the difference in subcellular localization may be linked to the cell type. Importantly, in normal human tissues and human tumors we detect nuclear, cytoplasmic and plasma membrane ARID3B [2] (data not shown). However the reasons for the differential localization are still under investigation. The membrane expression is not unique to ARID3B. ARID3A and ARID3C can also be found in lipid rafts when they are sumoylated [7]. ARID3B does not share the same conserved sumoylation site as ARID3A and ARID3C so the mechanism by which ARID3B is regulated to different compartments needs further investigation. Endogenous ARID3B Sh appears to be predominately nuclear soluble and bound to chromatin. However, when ARID3B Sh is overexpressed we observed an accumulation in the cytoplasm and mitochondria. Perhaps this is due to the fact that ARID3B Sh lacks a nuclear localization sequence and may require association with another protein that is a limiting factor in order to enter the nucleus.

In conclusion, our study demonstrated, for the first time, the presence of an alternate splice form of ARID3B and confirmed that both splice forms of ARID3B are present in a variety of cancer cell lines. We established that the EGFR modestly regulates ARID3B and this regulation may be assisted by the direct binding of PEA3 to the ARID3B promoter region. We also demonstrated that ARID3B splice forms are localized in different subcellular compartments. In addition, we made the novel observation, that ARID3B Fl induces apoptosis when overexpressed in ovarian cancer cells while ARID3B Sh does not. Although ARID3B is overexpressed in ovarian, we have demonstrated that one of the splice forms induces cell death. We predict that the subcellular localization of ARID3B may contribute to this discrepancy since ovarian tumors have high levels of cytoplasmic ARID3B Fl. Furthermore, we propose that ARID3B may be reminiscent of c-Myc that is both oncogenic and promotes apoptosis. Further work will address how localization and dosage of ARID3B regulates tumor cell growth and survival. These studies clearly identify a function for ARID3B Fl and it may be useful in increasing chemosensitivity in ovarian cancer cells and thereby improving therapeutic response.

## Materials and Methods

### Computational Analysis

An alternative splice form of ARID3B (ARID3B Sh) was identified through computational examination of the ARID3B locus. In brief, the cDNA sequence of ARID3B Fl was obtained from the National Center for Biotechnology Information (NCBI) website (http://www.ncbi.nlm.nih.gov). By inputting the ARID3B sequence into the University of California Santa Cruz (UCSC) Genome Browser website (http://genome.ucsc.edu) software, BLAT, unique expressed sequence tags (ESTs) of ARID3B were identified.

### Antibodies and Reagents

ARID3B antibodies for immunoblot and/or immunofluorescence analysis were purchased from Bethyl Laboratories, Inc. (Montgomery, TX): ARID3B (Cat# A302–564A: recognizing amino acids 100–150 present in both ARID3B Sh and ARID3B Fl) and ARID3B (Cat#A302–565A: recognizing amino acids 510–560 only present in ARID3B FL). We also generated an antibody to the unique epitope found only in ARID3B Sh (ECASTHHSNSGNTDRVPTVC) (Pacific Immunology, Ramona, CA).

For the western blots in Figures 2, 3, 5, 6, 7 and the Supplemental Figures Bethyl Laboratories ARID3B antibody: A302–564A), which recognizes both the ARID3B Fl and ARID3Sh isoforms was used. To confirm that this antibody recognized both isoforms we virally transduced OVCA 429 cells with ARID3B Sh or ARID3B Fl and isolated whole cell lysates for western blot analysis. Western blot performed using the ARID3B Fl specific antibody (Bethyl ARID3B Laboratories: A302–565A) (Figure S2A) detected a 61 kDa band. Western blot using an ARID3B Sh specific antibody (detects ARID3B Sh specific epitope (see above)) detected a 28 kDa band (Figure S2B). The Bethyl Laboratories ARID3B antibody (A302–564A), which recognizes amino acids 1–50 present in both ARID3B isoforms, identified bands for both ARID3B Fl and Sh at 61 kDa and 28 kDa (Figure S2C). Histone H3 was used as a loading control.

Other antibodies for immunoblot and/or immunofluorescence analysis included β-Actin, GAPDH, BIM, Caspase 7, Caspase 10, TNF-R2, TRADD and Histone H3 (Cell Signaling, Danvers, MA), Tom 20 (Santa Cruz Biotechnology, Inc., Santa Cruz, CA), Lamin B1 and TRAIL (Abcam, Cambridge, MA). Anti-rabbit Alexa Fluor 555 (Invitrogen, Carlsbad, CA) and Vectashield with 4′, 6-diamidino-2-phenylindole (DAPI) (Vector labs, Burlingame, CA) were used for immunofluorescence. PEA3 (Santa Cruz Biotechnology, Inc., Santa Cruz, CA) and IgG (Cell Signaling, Danvers, MA) were used for chromatin immunoprecipitation. All horseradish peroxidase conjugated secondary antibodies were purchased from GE Healthcare UK Ltd (Buckinghamshire, England). Annexin V-PE, Annexin V-FITC, and 7-Aminoactinomycin D (7-AAD) (eBiosceience, San Diego, CA) were used for flow cytometry analysis. A TNFα neutralizing antibody (Cell Signaling, Danvers, MA) was used for impairing TNFα activity. The following cell culture media and additives were obtained from Invitrogen (Carlsbad, CA): minimum essential media (MEM), RPMI, Dulbecco’s Modified Eagle Medium (DMEM), fetal bovine serum (FBS), penicillin/streptomycin, puromycin, blasticidin, 0.25% Trypsin-EDTA, L-glutamine and sodium pyruvate. F-12K media was obtained from American Type Culture Collection (ATCC) (Manassas, VA). Epidermal growth factor (EGF) and platelet-derived growth factor (PDGF) were purchased from R&D systems (Minneapolis, MN).

### Cell Culture

The ovarian epithelial carcinoma cell lines DOV13, OVCA429, and OVCA 433 were generously provided by Dr. Robert Bast, Jr., MD Andersen Cancer Center, Houston, TX [39], [40]. The ovarian cancer cell lines OVCA 433, OVCA 429 and DOV13 (and stable cell lines derived from these cells) were grown in Minimal essential media (MEM) supplemented 10% FBS, 0.1 mM L-glutamine, 1 mM sodium pyruvate, 50 U/ml penicillin, and 50 ug/ml streptomycin. The additional cancer cell lines were obtained from ATCC (Manassas, VA) and grown in the following media supplemented with 10% FBS, 50 U/ml penicillin, and 50 ug/ml streptomycin; breast cancer cell line: MCF-7 (MEM), pancreatic cancer cell line: BxPC-3 (RPMI), colon cancer cell line: SW 480 (RPMI), skin cancer cell line: A431 (DMEM), cervical cancer cell line: Ca Ski (RPMI) and prostate cancer cell line: PC3 (F-12K). All cell lines were propagated in a humidified incubator at 37°C with 5% CO_2_.

### Cell Transduction/Transfection

ARID3B Fl and ARID3B Sh were cloned into the lentiviral expression plasmid pLenti-suCMV-Rsv (RFP) (Gentarget, San Diego, CA). Enhanced green fluorescent protein (GFP) was expressed off the LV105 plasmid (GeneCopoeia, Rockville, MD). Virapower (Invitrogen, Carlsbad, CA) was used to package and produce lentiviral particles used to transduce cell lines. To generate ARID3B Sh, ARID3B Fl, and GFP expressing cells, ovarian cancer cell lines OVCA 433, OVCA 429 and DOV13 were transduced with lentiviral particles for ARID3B Sh, ARID3B Fl and GFP respectively for 72 h. Briefly, cells were grown to 40% confluency in MEM supplemented with 10% FBS, 0.1 mM L-glutamine, 1 mM sodium pyruvate, 50 U/ml penicillin, and 50 ug/ml streptomycin. After which time cells were transduced in MEM media containing 10% FBS, 0.1 mM L-glutamine, 1 mM sodium pyruvate, 5 µg/ml polybrene (Millipore, Billerica, MA) and with no penicillin or streptomycin. Stable cell lines were maintained in selection antibiotics blasticidin (ARID3B Sh) and puromycin (GFP) at concentrations ranging from 50 µg/ml to100 µg/ml.

### Western Blot Analysis

For the immunoblot analysis, whole-cell extracts were prepared by lysing cells in RIPA buffer (50 mM Tris pH 7.5, 150 mM NaCl, 1% NP-40, 0.5% EDTA, 0.1% SDS and protease inhibitor). Protein concentration was determined using the Bicinchoninic Acid (BCA) protein assay (Pierce, Rockford, IL). 30 µg of whole cell lysates was separated by SDS-PAGE and transferred to nitrocellulose membrane. Membranes were blotted with the following antibodies: ARID3B (Cat# A302–564A), ARID3B (Cat# A302–565A), ARID3B Sh, Caspase 7, Caspase 10, TNF-R2, TRADD, TRAIL, BIM, β-actin and GAPDH. Experiments were done in triplicates and densitometry analysis was performed using BIORAD Chemidoc XRS+ System using Imager Lab Software (Hercules, CA). The Imgenex Insta-blot ovarian tissue Oncopair was blotted for ARID3B (Bethyl Laboratories Cat# A302–564A) and stained with Ponceau S for loading. Statistics were calculated using student *t* test.

### Densitometry Analysis

Western blot imaging and densitometry analysis was performed using BIORAD Chemidoc XRS+ System using Imager Lab Software (Hercules, CA). Multiple exposures were captured for each blot and the system highlighted if the samples were oversaturated. In brief, densitometry was performed as follows. Rectangles of fixed area were drawn around western blot bands selected for analysis. Similar rectangles are drawn over regions of the western blot not containing bands and used determine the global background of the blot. The software determined the volume and area of the regions covered by the rectangles and subtracted the background volume from the volume of the western blot bands of interest. The same measurement of band intensity was done for loading controls (β-Actin, GAPDH or Histone H3). The loading control was used to normalize the relative values of western blot band. To calculate the relative intensity of each band, the intensity of the band was divided by the corresponding loading control intensity.

To validate the densitometry analysis, we ran different concentrations of cell lysates on SDS-PAGE gels and performed western blot analysis. Using Imager Lab Software we evaluated the western blots by densitometry analysis (Figure S2). Western blot analysis of OVCA 429 cells overexpressing ARID3B Sh demonstrated that by doubling the amount of protein [Lane 1 (20 µg protein): Lane 2 (40 µg protein)], the densitometry analysis reflects the 1∶2 ratio of protein present (Figure S2). The densitometry analysis was further confirmed in OVCA 429 cells overexpressing ARID3B Fl. Here we demonstrated that by tripling the amount of protein Lane 3 (20 µg protein): Lane 4 (60 µg protein)], the densitometry analysis reflects the 1∶3 ratio of protein present (Figure S2).

### Quantitative Real-time Polymerase Chain Reaction **(**QRT-PCR) Analysis

Total RNA was prepared from the samples using TRIzol (Invitrogen, Carlsbad, CA) according to the manufacturer’s protocol. Complementary DNA (cDNA) was synthesized (reverse transcribed polymerase chain reaction (RT-PCR)) from 0.4 µg of RNA using High Capacity cDNA Archive Kit according to the manufacturer’s protocol (Applied Biosystems, Carlsbad, CA). For QRT-PCR, SYBR master mix and Taqman master mix solution was obtained from Applied Biosystems (Carlsbad, CA) and primers for 18 S rRNA-conjugated with Texas Red (Millipore, Billerica, MA), 18 S rRNA, ARID3B Sh, ARID3B Sh-conjugated with HEX, TNFα, TNF-R2, TRAIL, TRADD, BCL10, BID, Caspase 10, Caspase 8, Caspase 7 were obtained from Integrated DNA Technologies (Coralville, Iowa) and ARID3B Fl-conjugated with FAM was obtained from Applied Biosystems (Carlsbad, CA). Conjugated primers were used in multiplex reactions. All samples were assayed in duplicated, and all experiments performed in triplicates using Biorad CFX96 C1000 System (Biorad, Hercules, CA). ΔΔC_T_ calculations were used to normalize signal versus18 S rRNA as the control. Statistics were calculated using student *t* test. QRT-PCR was performed using the following primers: ARID3B Sh-HEX: Primer 1: 5′- TCCCACCACATGGACAACAAGCTA-3′/Primer 2: 5′- TCATCCAGATTCCAGTTCGGCTGT 3′/HEX probe: 5′- AAGATGCTTCCAAGGCCTCACCTTCT-3′; ARID3B Fl-FAM-F, ARID3B Fl-FAM-R and FAM probe (Applied Biosystems, HS01084919_g1);

18 S rRNA-Texas red (Millipore, SCR595); ARID3B Sh: F: 5′- GACGGTGATCCTGAAAGGAA -3′/R: 5′- GAGGATTAGTGGGCCAGGAT-3′; 18 S rRNA: F: 5′- AAACGGCTACCACATCCAAG-3′/R: 5′-CCTCCAATGGATCCTCGTTA-3′; TNFα: primer 1: 5′-TTCGAGAAGATGATCTGATGC -3′/primer 2: 5′-TCAGCCTCTTCTCCTTCCT-3′; TRAIL primer 1: 5′- CTCAGGAATGAATGCCCACT-3′/primer 2: 5′-ACCAGAGGAAGAAGCAACAC -3′; TRADD: primer 1: 5′-GAGGACTCCACAAACAGGTATG-3′/primer 2: 5′-GGAGTAGAGCGGAGCCT -3′; TNF-R2: primer 1: 5′-CTCACAGGAGTCACACAC-3′/primer 2: 5′-CTATGACCAGACAGCTCAGATG -3′; Caspase 10: primer 1: 5′-TCCAGGCATGTCAGATTATCTTC-3′/primer 2: 5′-CCTTCTGAAAGACTCGCTTCC-3′; Caspase 8: primer 1: 5′-TCTTCAGCAGGCTCTTGTTG-3′/primer 2: 5′-AGAGGAAATCTCCAAATGCAAAC-3′; Caspase 7: primer 1: 5′-CGGGTGGTCTTGATGGA-3′/primer 2: 5′-AGATTCAGTGGATGCTCCGCC-3′; BCL10: primer 1: 5′-CTCAGCTATGATTTTCTCACACAG-3′/primer 2: 5′-CGGAAGAAGCGCCATCT-3′; BID: primer 1: 5′-TTCTCCATGTCTCTAGGGTAGG-3′/primer 2: 5′-GGACAGCATGGACCGTAG -3′.

Primer specificity for the ARID3B splice forms was addressed by overexpressing ARID3B Sh or ARID3B Fl in the ovarian cancer cell lines and harvesting the RNA for QRT-PCR analysis. The expression of ARID3B Fl significantly increased in ARID3B Fl overexpressing cells by 321- and 395- fold, however ARID3B Sh did not change significantly (1.2- and 1.1-fold) in OVCA 433 cells (Table 1) and OVCA 429 cells (Table 2), respectively. In cells overexpressing ARID3B Sh, the ARID3B Sh specific primer showed a significant increase in the expression of ARID3B Sh by 1907- and 3522- fold (OVCA 433 and OVCA 429 respectively). In cells overexpressing ARID3B Sh, ARID3B Fl increased by 5.2-fold (OVCA 433 cells, Table 1) and 4.8-fold (OVCA 429 cells, Table 2). These data suggest that ARID3B Sh primers are specific, but ARID3B Fl expression may be induced by ARID3B Sh. The data confirms the specificity of the primers for the ARIDB splice variants.

### Chromatin Immunoprecipitation (ChIP) Analysis

OVCA 429 cells were grown to 40% confluency, serum-starved in MEM containing 0.1% bovine serum albumin (BSA), 50 U/ml penicillin, 50 ug/ml streptomycin 0.1 mM L-glutamine and 1 mM sodium pyruvate for 24 h and treated with 20 nM of EGF for an additional 24 h. Using the Chip-IT Express Enzymatic kit (Active Motif, Carlsbad, CA) cells were prepared according to the manufacturer’s protocol. Immunoprecipitation (IP) was performed using anti-PEA3 and IgG antibodies (as a negative control). Input controls were also included (no antibody). Quantitative PCR was performed on the ARID3B promoter. As a negative control, we performed Quantitative PCR to an upstream region of the ETS binding sites in the ARID3B promoter. Quantitative PCR was performed using the following primers: ARID3B: F: 5′-ATACTCTCTCCTTTGCAGGCAGGT-3′/R: 5′-CTAACGTGTTCTGATCTCTCTTCC-3′; Upstream region of the ARID3B promoter: F: 5′-GCCTGGCCTTAACCTTTGTT-3′/R: 5′-CGATCTCCTGACCTCGTGAT-3′.

### Subcellular Fractionation Analysis

To analyze the intracellular localization of ARID3B, OVCA 429 ovarian cancer cells were transduced for 48 h as described above. Cells were serum-starved (as described above) and treated with 20 nM of EGF for an additional 24 h. Cells were harvested with 0.25% Trypsin-EDTA and washed twice with cold PBS. Subcellular protein fractions were prepared using the Subcellular Protein Fractionation Kit (ThermoScientific, Rockford, IL) according to the manufacturer’s instructions. The protein concentration was determined using the BCA protein assay and 10 µg of fractionated cell protein was used for western blot analysis as described above. Membranes were blotted with the following antibodies: ARID3B (Bethyl Laboratories Cat# A302–564A), Lamin B1, Histone H3 and Tom 20.

### Immunofluorescence Analysis

To further analyze the intracellular localization of ARID3B, OVCA 433 ovarian cancer cells were transduced as described above and plated in 4 well chamber slides. Cells were serum-starved as described above and treated with 20 nM EGF for an additional 24 h. Cells were washed thrice with 1X PBS and then fixed with ice-cold acetone. Tris-Buffered Saline and Tween 20 (TBST) (150 mM NaCl, 10 mM Tris pH 7.5 and 0.1% tween 20) permeablized cells were blocked with 3% BSA in TBST. Cells were incubated with a primary antibody recognizing both ARID3B isoforms (recognizing amino acids 100–150) diluted to 1∶600 in blocking buffer (TBST with 3% BSA) overnight at 4°C. The cells were rinsed thrice for 5 minutes with TBST, and incubated in secondary antibody (1∶1000; anti-rabbit Alexa Fluor 555) for 1 h at room temperature. Slides were again washed thrice for 5 minutes with TBST. The stained cells were mounted with Vectashield with DAPI and imaged using a Zeiss 710 Confocal Microscope (Carl Zeiss Microscopy, LLC, Thornwood, NY). Image J (NIH) was used to calculate the area and intensity of nuclei in cells that overexpress ARID3B Fl. The mean intensity (in arbitrary units) for each nucleus was normalized to the area of the nucleus. Student t test was used to determine the statistical significance of ARID3B immunofluorescence between ARID3B Fl expressing cells that were untreated or treated with 20 nM EGF for 24 h.

### FACScan Analysis of Apoptosis

For apoptosis studies subconfluent cells were either non-transduced or transduced with GFP, ARID3B Fl or ARID3B Sh lentiviral particles for 72 h as described above. Cells were harvested with 0.25% Trypsin-EDTA, washed twice with cold phosphate buffered saline (PBS) (137 mM M NaCl, 2.7 mM KCl, and 11.9 mM phosphate buffer, pH 7.4) and stained using either Annexin V-FITC or Annexin V-PE and 7-AAD per the manufacturer’s instructions. Annexin V and 7-AAD positively stained cells were assessed by flow cytometry using a Beckman Coulter FC500 Flow Cytometer (Brea, CA) and data was analyzed using WinMDI software (Scripps Research Institute, La Jolla, CA). Experiments were performed in triplicate and statistics were calculated using student *t* test.

### Caspase Activity Assay

Caspase 3/7 activity in OVCA 429 and OVCA 433 transduced cells (as described above) was measured using a Caspase-Glo 3/7 assay kit (Promega, Madison, WI) according to the manufacturer’s instructions in 96-well plates. In brief, OVCA 433 and OVCA 429 ovarian cancer cells were plated into 96-well plates (2000 cell per well), were grown overnight and then transduced for 72 h as described above. After transduction, cell culture media was changed and cells were grown for an additional 72 h. The luminescence of each sample was measured in a plate-reading luminometer and the data was handled with SoftmaxPro Version 3.1.1 (Molecular Devices, Sunnyvale, CA). Experiments were performed in triplicate, normalized to the number of cells and statistics were calculated using student *t* test.

### Cell Viability Assay

OVCA 433 and OVCA 429 ovarian cancer cells were plated into 96-well plates (2000 cell per well), were grown overnight and then transduced for 72 h as described above. After transduction, cell culture media was changed and cells were grown for an additional 72 h. Viable cells were assayed using the MTT (3-(4,5-Dimethylthiazol-2-yl)-2,5-diphenyltetrazolium bromide) cell proliferation assay according to the manufacturer*’*s instructions (Sigma). The plates were read at 595 nm, and the data were handled with SoftmaxPro Version 3.1.1 (Molecular Devices, Sunnyvale, CA). Experiments were performed in triplicate and statistics were calculated using student *t* test.

### Human Apoptosis Array

To determine the genes involved in ARID3B Fl induced apoptosis, total RNA was prepared from OVCA 429 cells transduced with ARID3B Fl or ARID3B Sh using TRIzol (Invitrogen, Carlsbad, CA) according to the manufacturer’s protocol. Complementary DNA (cDNA) was synthesized (reverse transcribed polymerase chain reaction (RT-PCR)) from 0.4 µg of RNA using High Capacity cDNA Archive Kit according to the manufacturer’s protocol (Applied Biosystems, Carlsbad, CA), followed by quantitative PCR-based human apoptotic gene array (TaqMan Human Apoptosis Array, Life Technologies, Grand Island, NY) according to the manufacturer’s protocol. Samples were assayed using Biorad CFX96 C1000 System (Biorad, Hercules, CA). ΔΔC_T_ calculations were used to normalize signal versus 18 S rRNA as the control. A complete list of 96 apoptosis-related genes included in the analyses and detailed protocols can be found at the manufacturer’s web site: (http://products.invitrogen.com/ivgn/product/4414072).

### Human TNFα Immunoassay

The levels of TNFα in cell culture supernatant in OVCA 429 and OVCA 433 transduced cells (as described above) were measured using Human TNFα Immunoassay (R&D Systems, Inc., Minneapolis, MN). In brief, 1×10^4^ cells per well were plated in 12-well plate, cells were transduced and after three days the media changed. After an additional three days the cell culture supernatant was collect. Cell culture supernatants were analyzed according to the manufacturer’s instructions. The plates were read at 405 nm and 540 nm (for wavelength correction), and the data were handled with SoftmaxPro Version 3.1.1 (Molecular Devices, Sunnyvale, CA). Experiments were performed in triplicate and statistics were calculated using student *t* test.

### Treatment of ARID3B Fl Ovarian Cancer Cells with TNFα Neutralizing Antibody

To determine if the TNFα pathway contributed to ARID3B Fl-induced apoptosis, we utilized an anti-TNFα neutralizing antibody. OVCA 429 ovarian cancer cells were treated with or without 10 µg/ml of anti-TNFα antibody, 24 h prior to transducing cells with ARID3B Fl as described above. Seventy-two hours post transduction the cells were harvested and analyzed for apoptotic activity via flow cytometry as described above (FACS Analysis of Apoptosis).

### Statistical Analysis

Experimental values were expressed as mean ± SEM. Differences between mean values for QRT-PCR, ELISA, Caspase-Glo 3/7, MTT, Q-PCR (ChIP) and densitometry, analysis were evaluated using Student’s t-test (GraphPad Software, Inc., La Jolla, CA); p<0.05 was considered statistically significant.

## Supporting Information

Figure S1 **ARID3B is expressed in ovarian tumors but not normal adjacent tissue.** Western blot was performed on Imgenex Insta-blot ovarian tissue Oncopair for ARID3B. This demonstrates ARID3B Fl is expressed in 7/7 serous tumors but not in normal adjacent tissue lysates. ARID3B was present in the tumors lysates and in some normal adjacent tissue lysates. *Denotes location of ARID3B Fl (61 kDa) and ARID3B Sh (28 kDa). Ponceau S staining of blot was used as a loading control.
(TIF)

Figure S2 **Validation of ARID3B Isoform antibodies.** Western blot analysis from whole cell lysates from OVCA 429 cells overexpressing ARID3B Sh and ARID3B Fl was performed for both splice forms using the ARID3B antibodies specific to different regions of ARID3B and Histone H3 (loading control). (**A**) An anti-ARID3B Fl antibody (Bethyl Laboratories ARID3B antibody: A302–565A), which recognizes amino acids 510–560 only present in ARID3B Fl detects ARID3B Fl at 61 kDa. (**B**) An anti-ARID3B Sh antibody, which recognizes the unique epitope found only in ARID3B Sh detects a band specifically at 28 kD. (**C**) Western blot using the anti-ARID3B antibody (Bethyl Laboratories ARID3B: A302–564A) which recognizes both the ARID3B Fl and ARID3B Sh (amino acids 100–150) detects bands at 61 and 28 kDa. The densitometry evaluation of the western blot analyses for ARID3B isoforms was analyzed (value under blot). *ARID3B Sh overexpressing cell lysate: 20 µg [lanes 1] and 40 µg [lanes 2] and ARID3B Fl overexpressing cell lysate: 20 µg [lanes 3] and 60 µg [lanes 4.].*
(TIF)

Figure S3 **Subcellular localization of overexpressed ARID3B splice forms.** OVCA 429 cells transduced with ARID3B Fl (**A**) or ARID3B Sh (**B**) were serum starved (SS) for 24 h, treated with or without 20 nM EGF for 24 h, and fractionated into cytoplasmic, membrane, nuclear and chromatin-bound extract fractions. Western blot was performed for ARID3B. Uncropped blots are provided to demonstrate the level of overexpression seen in the transduced cell used in the fractionation studies. Cytoplasmic fraction [lanes 1 (no EGF) and 2 (20 nM EGF)], membrane fraction [lanes 3 (no EGF) and 4 (20 nM EGF)], nuclear soluble fraction [lanes 5 (no EGF) and 6 (20 nM EGF)] and chromatin-bound fraction [lanes 7 (no EGF) and 8 (20 nM EGF)].
(TIF)

Table S1 **Genes differentially regulated by the overexpression of ARID3B Fl**
(DOC)
