# Supplementary figures and images for: ARID3B Induces Tumor Necrosis Factor Alpha Mediated Apoptosis While a Novel ARID3B Splice Form Does Not Induce Cell Death
Source: PLoS One. 2012 Jul 31;7(7):e42159. doi: 10.1371/journal.pone.0042159 (PMC3409141; doi:10.1371/journal.pone.0042159)

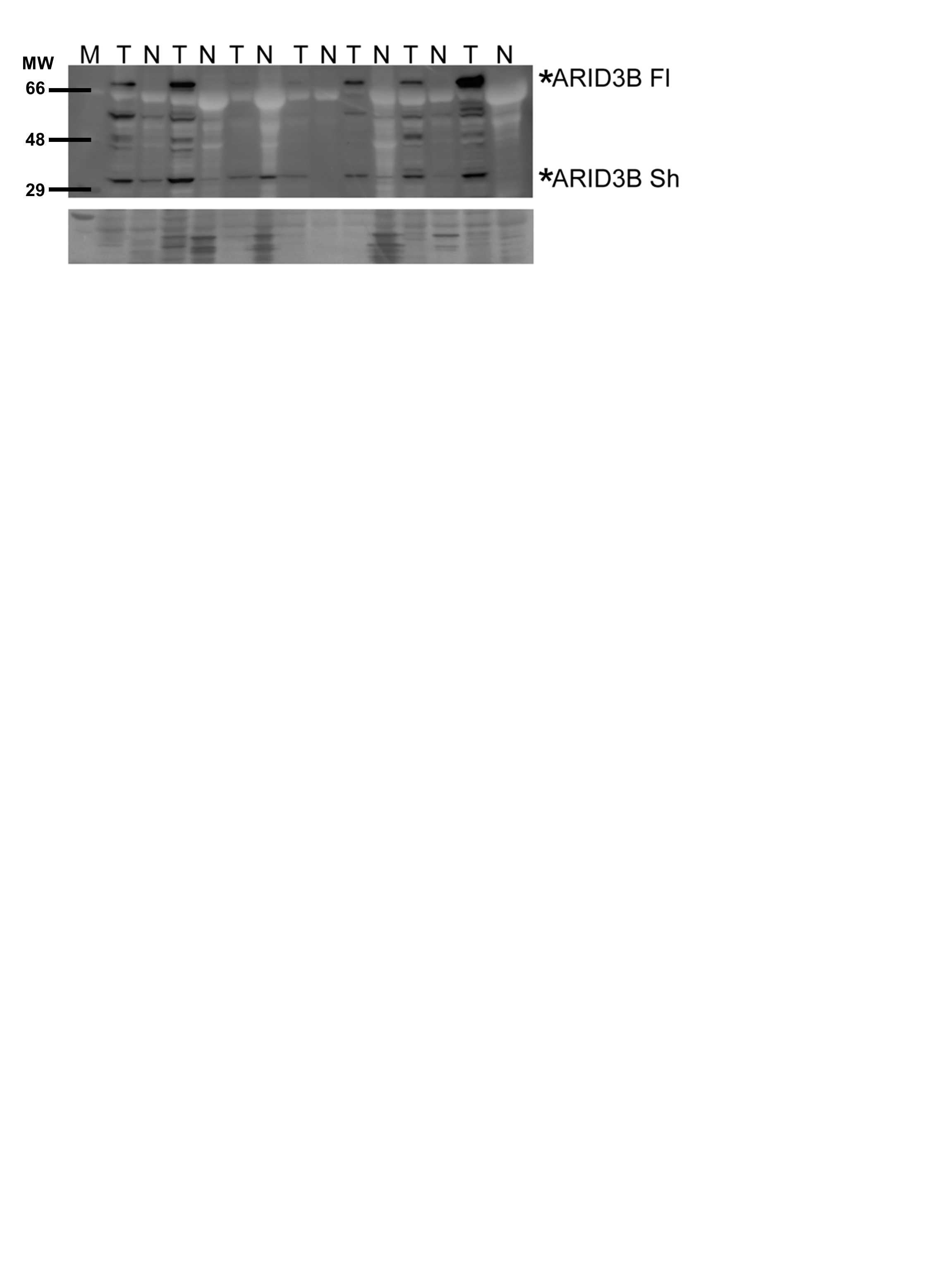

Supplement: Figure S1 — ARID3B is expressed in ovarian tumors but not normal adjacent tissue. Western blot was performed on Imgenex Insta-blot ovarian tissue Oncopair for ARID3B. This demonstrates ARID3B Fl is expressed in 7/7 serous tumors but not in normal adjacent tissue lysates. ARID3B was present in the tumors lysates and in some normal adjacent tissue lysates. *Denotes location of ARID3B Fl (61 kDa) and ARID3B Sh (28 kDa). Ponceau S staining of blot was used as a loading control. (TIF) [file pone.0042159.s001.tif]

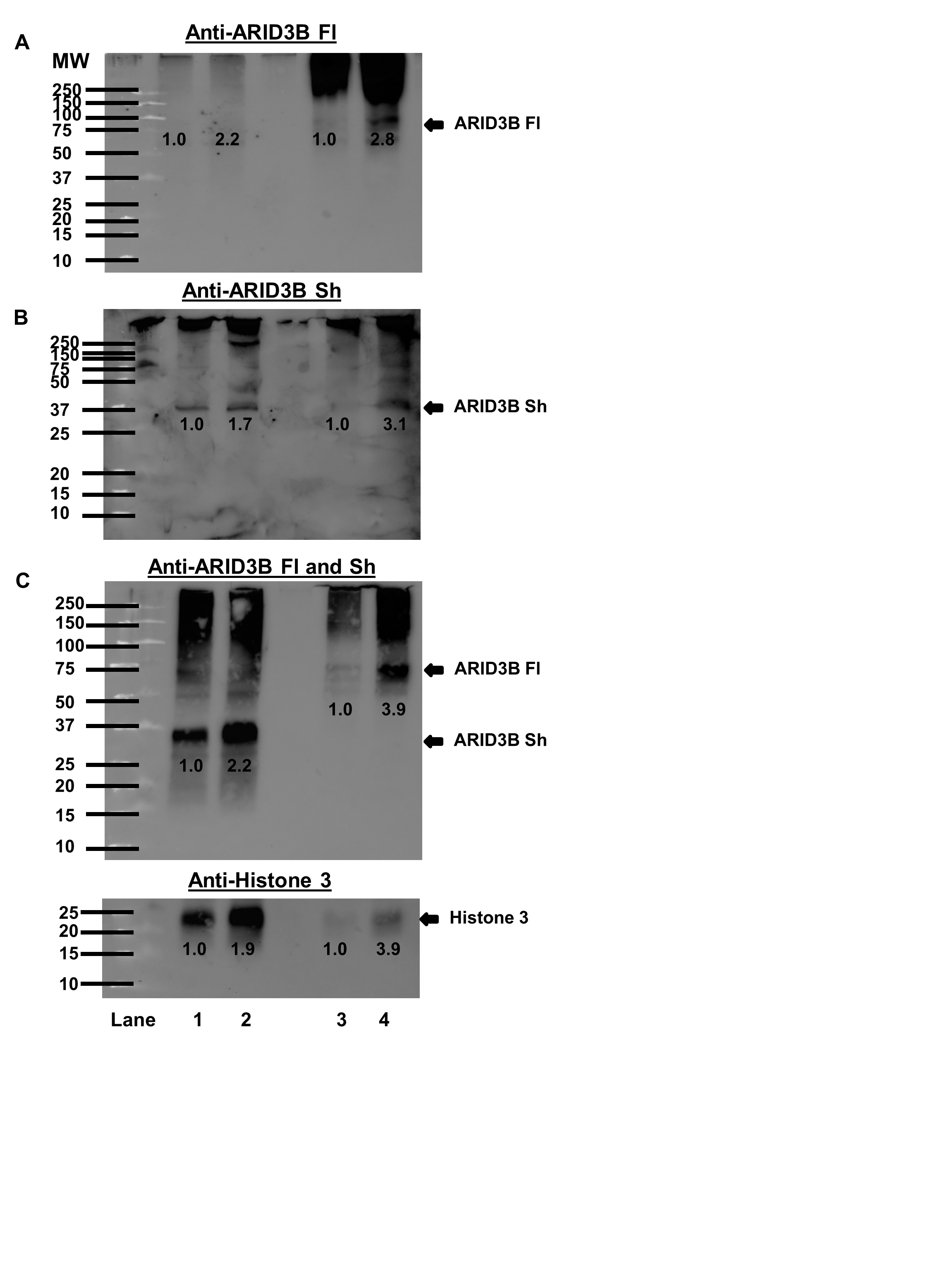

Supplement: Figure S2 — Validation of ARID3B Isoform antibodies. Western blot analysis from whole cell lysates from OVCA 429 cells overexpressing ARID3B Sh and ARID3B Fl was performed for both splice forms using the ARID3B antibodies specific to different regions of ARID3B and Histone H3 (loading control). (A) An anti-ARID3B Fl antibody (Bethyl Laboratories ARID3B antibody: A302–565A), which recognizes amino acids 510–560 only present in ARID3B Fl detects ARID3B Fl at 61 kDa. (B) An anti-ARID3B Sh antibody, which recognizes the unique epitope found only in ARID3B Sh detects a band specifically at 28 kD. (C) Western blot using the anti-ARID3B antibody (Bethyl Laboratories ARID3B: A302–564A) which recognizes both the ARID3B Fl and ARID3B Sh (amino acids 100–150) detects bands at 61 and 28 kDa. The densitometry evaluation of the western blot analyses for ARID3B isoforms was analyzed (value under blot). ARID3B Sh overexpressing cell lysate: 20 µg [lanes 1] and 40 µg [lanes 2] and ARID3B Fl overexpressing cell lysate: 20 µg [lanes 3] and 60 µg [lanes 4.]. (TIF) [file pone.0042159.s002.tif]

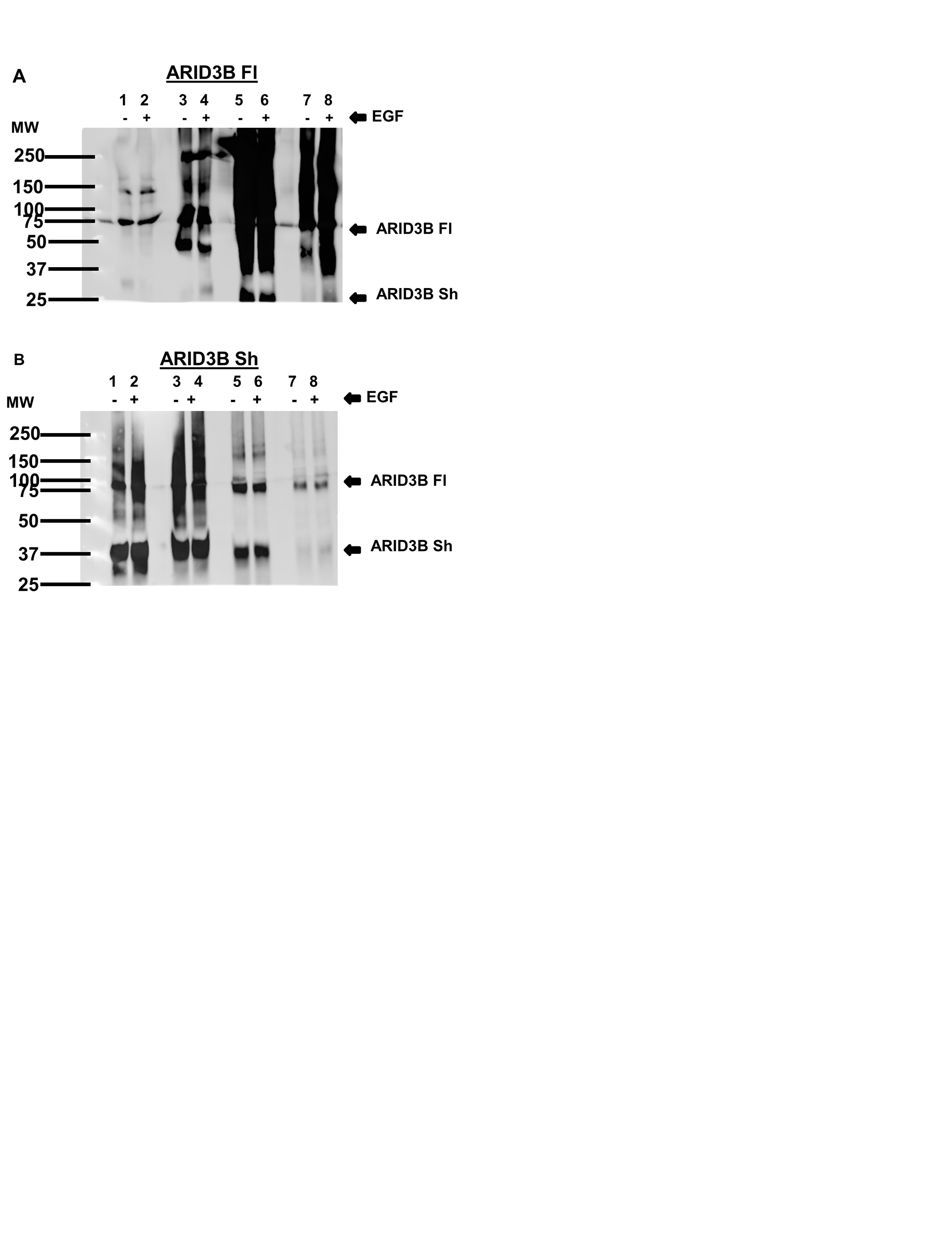

Supplement: Figure S3 — Subcellular localization of overexpressed ARID3B splice forms. OVCA 429 cells transduced with ARID3B Fl (A) or ARID3B Sh (B) were serum starved (SS) for 24 h, treated with or without 20 nM EGF for 24 h, and fractionated into cytoplasmic, membrane, nuclear and chromatin-bound extract fractions. Western blot was performed for ARID3B. Uncropped blots are provided to demonstrate the level of overexpression seen in the transduced cell used in the fractionation studies. Cytoplasmic fraction [lanes 1 (no EGF) and 2 (20 nM EGF)], membrane fraction [lanes 3 (no EGF) and 4 (20 nM EGF)], nuclear soluble fraction [lanes 5 (no EGF) and 6 (20 nM EGF)] and chromatin-bound fraction [lanes 7 (no EGF) and 8 (20 nM EGF)]. (TIF) [file pone.0042159.s003.tif]
