# Supplementary material for: ARID3B Induces Tumor Necrosis Factor Alpha Mediated Apoptosis While a Novel ARID3B Splice Form Does Not Induce Cell Death
Source: PLoS One. 2012 Jul 31;7(7):e42159. doi: 10.1371/journal.pone.0042159 (PMC3409141; doi:10.1371/journal.pone.0042159)
Supplement: Table S1 — Genes differentially regulated by the overexpression of ARID3B Fl (DOC) [file pone.0042159.s004.doc]

| **Table S1. Genes differentially regulated by the overexpression of ARID3B Fl** | | | |
| --- | --- | --- | --- |
| Gene | Fold Expression | Gene | Fold Expression |
| 18S | 1.0 | NLRP1 | 1.5 |
| XIAP | 0.7 | BCL2L11 | 1.5 |
| Bcl-2 | 0.1 | LTA | 1.5 |
| Bcl-xL | 0.8 | CASP7 | 1.5 |
| IKBKE | 1.1 | NAIP | 1.6 |
| BNIP3L | 1.1 | TNFRSF1A | 1.6 |
| BAD | 1.1 | BID | 1.7 |
| BCAP31 | 1.1 | CASP10 | 1.7 |
| DEDD2 | 1.1 | BIRC8 | 2.38 |
| REL | 1.2 | TNFSF10 | 2.8 |
| BOK | 1.2 | CARD9 | 4.5 |
| CASP1 | 1.2 | LTB | 1.1x10^1^ |
| BCL3 | 1.2 | BCL10 | 2.5x10^1^ |
| IFT57 | 1.2 | TNFRSF1B | 2.7x10^1^ |
| HIP1 | 1.3 | TNF | 3.8x10^1^ |
| HPRT1 | 1.3 | BIRC7 | 3.2x10^2^ |
| BAK1 | 1.3 | TRADD | 1.1x10^9^ |
| NFKBIA | 1.4 |  |  |
| ΔC_T_ calculations were analyzed by comparing C_T_ value of ARID3B Fl and ARID3B Sh transduced samples of a specific gene target. ΔΔC_T_ calculations were used to normalize signal versus 18S rRNA as the control. (N=1) | | | |
